# Distinct response properties between the FFA to faces and the PPA to houses

**DOI:** 10.1002/brb3.2706

**Published:** 2022-07-18

**Authors:** Mengjin Li, Hong Huang, Bingbing Guo, Ming Meng

**Affiliations:** ^1^ Philosophy and Social Science Laboratory of Reading and Development in Children and Adolescents (South China Normal University) Ministry of Education Guangzhou China; ^2^ Guangdong Key Laboratory of Mental Health and Cognitive Science, School of Psychology South China Normal University Guangzhou China; ^3^ School of Teacher Education Nanjing Xiaozhuang University Nanjing China

**Keywords:** category selectivity, cortical organization, face perception, fMRI, response properties

## Abstract

**Introduction:**

The object recognition system involves both selectivity to specific object category and invariance to changes in low‐level visual features. Mounting neuroimaging evidence supports that brain areas in the ventral temporal cortex, such as the FFA and PPA, respond preferentially to faces and houses, respectively. However, how regions in human ventral temporal cortex partitioned and functionally organized to selectively and invariantly respond to different object categories remains unclear. What are the changes of response properties at the intersection of adjacent but distinctively‐selective regions?

**Method:**

Here, we conducted an fMRI study and three‐pronged analyses to compare the brain mapping relationships between the FFA to faces and the PPA to houses. Specifically, we examined: 1) the response properties of object selectivity to the preferred category; 2) the response properties of invariance to contrast and a concurrently presented non‐preferred category; 3) whether there are asymmetrical changes of response properties across the boundary from the FFA to PPA versus from the PPA to FFA.

**Results:**

We found that the response properties of FFA are highly selective and reliably invariant, whereas the responses of PPA vary with the image contrast and concurrently presented face. Moreover, the response properties across the boundary between the FFA and PPA are asymmetrical from face‐selective to house‐selective relative to from house‐selective to face‐selective.

**Conclusions:**

These results convergently revealed distinct response properties between the FFA to faces and the PPA to houses, implying a combination of spatially discrete domain‐specific and relatively distributed domain‐general organization mapping in human ventral temporal cortex.

## Graphical abstract

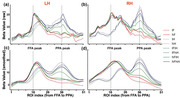

## 1 INTRODUCTION

Object recognition is a fundamental visual cognition ability. At any given moment, our visual environment normally contains multiple objects. We recognize these objects quickly and effortlessly even when they are encountered in unusual orientations, under different illumination conditions, or partially occluded by other objects. Extensive human and nonhuman primate studies have identified putative higher‐level visual areas in ventral temporal cortex that mediate object recognition. Neurons from distinct clusters in ventral temporal cortex differentially and selectively activate to specific object category (Grill‐Spector & Weiner, 2014; Haxby, Gobbini, et al., 2001; Kanwisher & Yovel, 2006; Kanwisher et al., 1997). For example, the activity of the fusiform face area (FFA) is highly specific to faces and face‐like stimuli, whereas the parahippocampal place area (PPA) responds selectively to houses (or scenes, landmarks, cityscapes, and rooms) (Epstein & Baker, 2019; Epstein & Kanwisher, 1998; Kanwisher et al., 1997). These functionally specialized, category‐selective regions are widely assumed to provide object selectivity and to a large extent tolerant to confounding factors such as object size, position, illumination, and other objects that were concurrently presented (Bao & Tsao, 2018; Logothetis & Sheinberg, 1996; Quiroga et al., 2005; Tanaka, 1996).

However, despite being extensively examined, how regions in human ventral temporal cortex are partitioned and functionally organized to respond selectively to specific category and invariantly to other object categories remains controversial (Grill‐Spector & Weiner, 2014; Haxby et al., 2001; Quiroga et al., 2005; Tsao & Livingstone, 2008; Zoccolan et al., 2005, 2007). A large body of studies emphasized that neurons in temporal cortex were both highly selective and reliably invariant (Andrews & Ewbank, 2004; Grill‐Spector et al., 1999; Quiroga et al., 2005; Rust & Dicarlo, 2010). Responses of category‐selective areas to a simultaneously presented object pair were similar to those to the preferred category in isolation (i.e., a max response in face areas to faces or in scene areas to scenes) (Bao & Tsao, 2018; Reddy & Kanwisher, 2007). On the other hand, some studies suggested a trade‐off relationship between object selectivity and low‐level features invariance. That is, neurons with high object selectivity typically have relatively low invariance and vice versa (Zoccolan et al., 2007). Responses to the preferred object were often reported to reduce by the presence of a nonpreferred object in category‐selective areas, and responses to a pair of objects were very close to the average of responses to each object individually (Baeck et al., 2013; Kliger & Yovel, 2020; MacEvoy & Epstein, 2009; Zoccolan et al., 2005). Finally, a compromising hypothesis holds that both types of information are available from different neuronal populations in the temporal cortex, namely, information about specific object features like retina location and size, as well as information about object identity, which is tolerant to changes in low‐level visual features (Lueschow et al., 1994; Hung et al., 2005; Guo & Meng, 2015; Kay & Yeatman, 2017; Schwarzlose et al., 2008; Yue et al., 2011).

Here, we examined fMRI responses on a fine‐scale across relatively extensive cortical surface areas in ventral temporal cortex, including the FFA and PPA, to investigate the response properties of category selectivity to the preferred stimulus and invariance to changes in image contrast and a concurrently presented nonpreferred stimulus. Among all different types of objects, faces may be a unique category given their evolutionarily crucial role in normal human social interactions. Much evidence supports prioritized processing of faces and the specific cognitive and neural machinery of face processing, which cannot be shared by other kinds of object perception (Duchaine & Yovel, 2015; Kanwisher & Yovel, 2006; Tsao et al., 2003, 2006; Tsao & Livingstone, 2008). It is possible that the FFA would be more selective to faces than other regions selective to their preferred category (e.g., PPA to houses), and thus the response of FFA would be uniquely and reliably tolerant to changes in low‐level visual features as well as to concurrently presented nonpreferred objects (e.g., houses). However, if a common rule were applied to fairly partition regions in ventral temporal cortex according to their preferred categories, there should be similar response patterns between, for example, how the FFA responds to faces and how the PPA responds to houses.

Moreover, whether there is a clear boundary or a symmetrical change in response properties between two adjacent but distinctively‐selective regions (i.e., FFA and PPA) remains elusive. Measurements in the primate inferior temporal cortex have shown that the proportion of selective neurons for a particular category within a category‐selective region, which was detected by using fMRI, is significantly higher than the outside of this region. Within fMRI identified category‐selective regions in temporal cortex, a wide range of 29–97% neurons are selective for the preferred category, with the highest proportion in face‐selective patches (Bell et al., 2011; Issa et al., 2013; Tsao et al., 2006). However, as the FFA and PPA are adjacent to each other, neuron selectivity across the boundary between the FFA and PPA remains unknown. According to the category specialized modular hypothesis, faces and houses are represented and discriminated in functionally specialized and spatially distinct cortical areas. Thus, there should be a clear boundary of neural response properties between the FFA to faces and the PPA to houses. In contrast, distributed theories argue that category‐specific patterns of response are widely distributed and overlapping organized (Behrmann & Plaut, 2013; Bell et al., 2011; Cohen & Tong, 2001; Cox & Savoy, 2003; Haxby, Gobbini, et al., 2001; Weiner & Grill‐Spector, 2010). Thus, it may be hard to discern the boundary between two adjacent category‐selective regions in ventral temporal cortex, including the FFA and PPA.

In the present study, we specified multiple successive regions of interest (ROIs) on a fine‐scale along a virtual line connecting the FFA and PPA on the inflated gray matter surface to investigate response patterns of two adjacent regions. Responses of defined ROIs to image contrasts of faces and houses as well as concurrently presented face‐house stimuli were examined. In addition, we also compared variations in category selectivity and invariance across the boundary between the FFA and PPA. We hypothesize that, if category‐selective regions might be modularly partitioned and functionally organized at the same level, changes in face‐selectiveness versus house‐selectiveness across the boundary between the FFA and PPA should be symmetrical. If how the FFA responds to faces might be different from how the PPA responds to houses, we should observe asymmetrical changes in face‐selectiveness versus house‐selectiveness across the boundary from the FFA to PPA and vice versa from the PPA to FFA.

## 2 MATERIALS AND METHODS

### 2.1 Subjects

Seventeen healthy adults participated in this study with informed consent, including seven females, on average 26 years old. All subjects had normal or corrected‐to‐normal visual acuity and color vision. This study was approved by the Dartmouth College Human Protection Committee. Data from five subjects with head movements greater than 3 mm were excluded from further data analyses. Part of the data was independently analyzed and published for a separate study (Guo & Meng, 2015).

### 2.2 Materials

**FIGURE 1:**
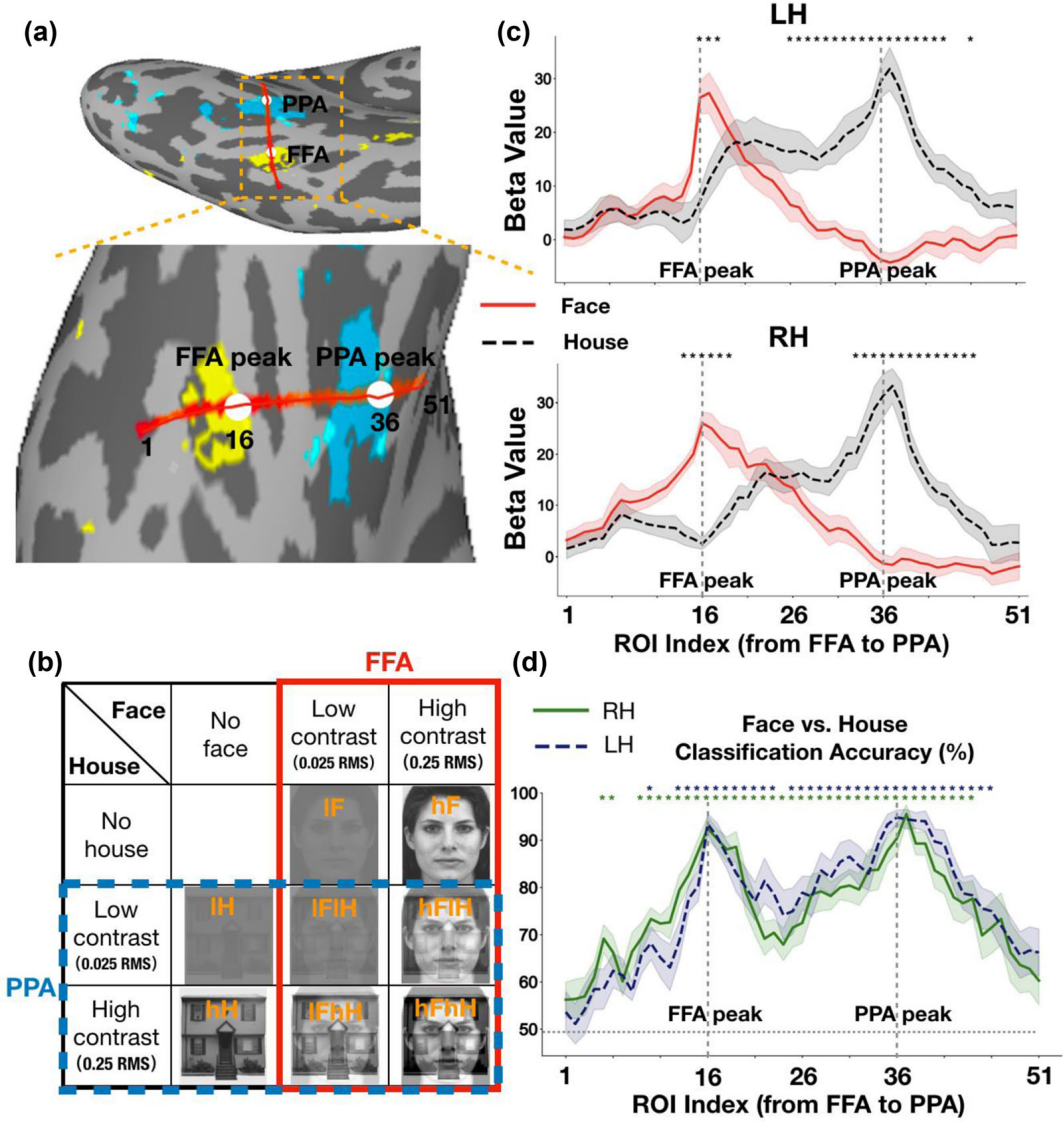
(a) Schematic visualization of the functional ROIs from a representative subject. Each region was defined individually for each subject from their functional localizer scans. The FFA (yellow), PPA (cyan), and the virtual line (red) connecting the FFA and PPA peak foci (white dots) are shown on the inflated surface. (b) Examples of stimuli and experimental conditions in the main experiment. The red box (solid line) represents a two‐way factorial design (two levels of face contrast (low (lF, lFlH, and lFhH) vs. high (hF, hFlH, and hFhH)) × three levels of house contrast (none (lF and hF) vs. low (lFlH and hFlH) vs. high (lFhH and hFhH))) for the FFA selective to faces. The blue box (dashed line) represents a separate two‐way factorial design (two levels of house contrast (low (lH, lFlH, and hFlH) vs. high (hH, lFhH, and hFhH)) × three levels of face contrast (none (lH and hH) vs. low (lFlH and lFhH) vs. high (hFlH and hFhH))) for the PPA selective to houses. (c and d) Univariate and multivariate results of the localizer experiment in ROIs along the virtual line from the FFA to PPA. (c) Results of BOLD responses to faces and houses. The solid red line represents the face condition and the dashed black line represents the house condition. (d) Results of classification accuracy for distinguishing between faces and houses. The dashed blue line represents results of the left hemisphere and the solid green line represents results of the right hemisphere. Shaded areas represent ± 1 S.E.M. and significances are marked with stars (*p*s < .001, corrected). LH is the abbreviation for the left hemisphere and RH is the abbreviation for the right hemisphere

The stimuli set of the localizer experiment was grayscale images, including 16 faces and 16 houses. An independent set of grayscale images was collected for the main experiment, including eight faces and eight houses. All stimuli images of the main experiment were equally transformed into a high‐contrast version equivalent to 0.25 root mean square (RMS) and a low‐contrast version equivalent to 0.025 RMS in normalized units. The contrast of all stimuli conditions was adjusted by using MATLAB and the SHINE toolbox (Willenbockel et al., 2010). In the main experiment, there were eight experimental conditions, including four single‐image conditions and four face‐house overlapping‐image conditions: low‐contrast faces with 0.025 RMS (lF), high‐contrast faces with 0.25 RMS (hF), low‐contrast houses with 0.025 RMS (lH), high‐contrast houses with 0.25 RMS (hH), overlapping images of a low‐contrast face with 0.025 RMS superimposed on a low‐contrast house with 0.025 RMS (lFlH), overlapping images of a low‐contrast face with 0.025 RMS superimposed on a high‐contrast house with 0.25 RMS (lFhH), overlapping images of a high‐contrast face with 0.25 RMS superimposed on a low‐contrast house with 0.025 RMS (hFlH), and overlapping images of a high‐contrast face with 0.25 RMS superimposed on a high‐contrast house with 0.25 RMS (hFhH) (Figure 1(b)). The stimuli were presented in the center of the screen and the visual angle of stimuli was 8.7^o^.

### 2.3 MRI acquisition

Image acquisition was performed at Dartmouth Brain Imaging Center using a Philips Achieva Intera 3.0 T scanner with a 32‐channel head coil. Anatomical T1‐weighted 3D images were acquired using a magnetization‐prepared rapid‐acquisition gradient echo sequence at the end of the scan session for each subject (TR = 8.2 ms, TE = 3.8 ms, flip angle = 8°, FOV = 240 mm, voxel size = 1 × 1 × 1 mm, 222 slices). BOLD images were collected using an echo‐planar imaging (EPI) sequence (TR = 2000 ms, TE = 35 ms, flip angle = 90°, FOV = 240 mm, voxel size = 3 × 3 × 3 mm, 35 slices). During EPI scans, visual stimuli were presented to subjects via a Panasonic PT‐D4000U projector using MATLAB with Psychtoolbox (Brainard & Freeman, 1997; Pelli, 1997). The rear‐projection screen was positioned at the rear of the scanner and viewed with a mirror mounted to the head coil. The width and height of the projected screen were 45.7 and 34.3 cm (1024 × 768 pixels), respectively. The distance between the mirror and the projected screen was 97.8 cm and the distance between the mirror and subjects’ eyes was approximately 12.7 cm.

### 2.4 Experiment procedures

In the localizer experiment, each participant performed two runs to localize the FFA and PPA as ROIs. Each run was consisted of five face blocks and five house blocks, by alternating presenting, where each run started with a 16 s fixation block. Each stimulation block was 16 s, followed by a 16 s fixation period. Within each block there were 16 images from a single stimulus condition, and each image was presented for 500 ms with a 500 ms inter‐stimulus‐interval (ISI). The participant performed a one‐back repetition detection task to press a button whenever there was a repetition of an image.

In the main experiment, we presented a participant with eight different types of visual stimuli (lF, hF, lH, hH, lFlH, lFhH, hFlH, hFhH) in a block design. The participant performed a color detection task to press a button whenever the whole image turned to red for 200 ms at random times during the stimulus presentation. This study was consisted of nine or ten runs, where each run was comprised of eight blocks, one for each stimulus condition. Each run started with a 16 s fixation block, and each stimulation block was interleaved with a 16 s fixation block. Within each block there were eight images from a single stimulus condition, and each image was presented for 1700 ms with a 300 ms ISI. In total, each stimulation block was 16 s, and each run was 272 s. The blocks were randomized across each run.

### 2.5 Data analysis

#### 2.5.1 Preprocessing

T1‐weighted images were processed using FreeSurfer's (Fischl, 2012) recon‐all processing pipeline to segment subcortical structures and reconstruct the cortical surface. The functional data were processed with a surface‐based processing pipeline using AFNI and SUMA. For each BOLD run, we conducted the following processing: remove spikes using *3dDespike*, slice‐time correction using *3dTshift*, compute anatomical alignment transformation to EPI registration using *align_epi_anat*, head motion correction using *3dvolreg* and normalization using *3dcalc* (Cox, 1996; Saad & Reynolds, 2012). BOLD images were motion corrected and aligned to the first volume of the first run. Data with equal or greater than 5 mm motion in any direction were excluded from further analysis. Using this criterion, five subjects was excluded. As we have reconstructed the cortical surfaces, we can use *@SUMA_Make_Spec_FS* to create a new surface that can be read by SUMA, use *@SUMA_AlignToExperiment* to create a version of surface anatomy that has been registered to the experiment anatomical volumes, and use *3dVol2Surf* to map data values from AFNI volume dataset to the surface dataset. Then, surface‐based images were submitted to a general linear model (GLM using 3dDeconvolve to obtain the beta coefficient values associated with each block for each condition. All data were analyzed in both the native space of each observer and the MNI305 space. The localizer data and the main experiment data were processed in the same way.

#### 2.5.2 ROIs localization from the FFA to PPA

To measure the changes of brain activity for faces and houses across continuum regions from the FFA to the PPA, we specified a virtual line connecting the FFA and PPA in the ventral temporal cortex on the inflated gray matter surface for each subject (Figures 1(a) and S1). First, to functionally localize the FFA and PPA as ROIs, a whole‐brain GLM analysis was performed by contrasting faces versus houses on the localizer data for each subject separately. Vertices were selected among several FreeSurfer parcellations, including parahippocampal gyrus, collateral sulcus, lingual sulcus, fusiform gyrus, inferior temporal sulcus, lateral occipito‐temporal sulcus, inferior temporal gyrus, and posterior transverse collateral sulcus (aparc. A2009s/Destrieux.simple.2009‐07‐29.gcs atlas). Vertex with the strongest response to faces was selected as the FFA peak foci and vertex with the strongest response to houses was selected as the PPA peak foci within the aforementioned masks. For each subject, corresponding RAS coordinates of the selected FFA peak vertex and PPA peak vertex were obtained from the inflated gray matter surface. Then we transformed them into MNI surface coordinate system by convolving the native matrix with Talairach transformation matrix. Next, we specified a virtual line connecting the coordinate of FFA peak foci (ROI index 16) and the coordinate of PPA peak foci (ROI index 36) on the inflated gray matter surface. Nineteen sets of coordinates (ROI index 17–35) were selected with equal spacing in‐between along the virtual line. Then, we extended the virtual line by 15 sets of coordinates (ROI index 1–15 and ROI index 37–51) with identical space to both sides. For each set of coordinates, we grouped five coordinates closest to the given coordinate as one ROI (index) according to Euclidean distance. In total, there were 51 sets of ROIs along the line connecting the FFA and PPA for each hemisphere. Finally, we get the corresponding nodes index of the selected 51 sets of ROIs on the inflated surface. Coordinates of the FFA peak foci, the PPA peak foci and two endpoints of the virtual line has been displayed in the supplementary materials for each subject (Table S13). The size of each ROI was about 2.874 mm^2^ on average. The cortical surface distance between the FFA peak and PPA peak was about 42.51 mm for the left hemisphere and 40.34 mm for the right hemisphere on average.

#### 2.5.3 ROIs localization from the occipital pole to the FFA and from the occipital pole to the PPA

To investigate the brain selectivity for faces and houses from the occipital lobe to the ventral temporal cortex, we specified two virtual lines on the inflated gray matter surface from the occipital pole to the FFA and from the occipital pole to the PPA. We first selected the median coordinate of the occipital lobe (ROI index 16) as one starting point of the virtual line according to the anatomical mask in FreeSurfer. The selected coordinates of the occipital pole in the right hemisphere were [ −20, −125, −41], and those in the left hemisphere were [25, −123, −54] in the MNI surface coordinate system. Then, 28 sets of coordinates (ROI index 17–44) were selected with equal spacing in‐between along the virtual line from the occipital pole (ROI index 16) to the FFA/PPA peak (ROI index 45). The virtual line was extended by 15 sets of coordinates (ROI index 1–15 and ROI index 46–60) with identical space to both sides. Similarly, for each set of coordinates, we grouped 5 coordinates closest to the given coordinate as one ROI (index) according to Euclidean distance. In total, there were 60 sets of ROIs along the two virtual lines connecting the occipital pole and the FFA/PPA for each hemisphere. Finally, we get the corresponding nodes index of the selected 60 sets of ROIs on the inflated surface. The cortical surface distance between the occipital pole and the FFA peak was about 72.55 mm for the left hemisphere and 75.17 mm for the right hemisphere on average. The cortical surface distance between occipital pole and the PPA peak was about 73.80 mm for the left hemisphere and 74.46 mm for the right hemisphere on average.

#### 2.5.4 Univariate analysis of category selectivity

**FIGURE 2:**
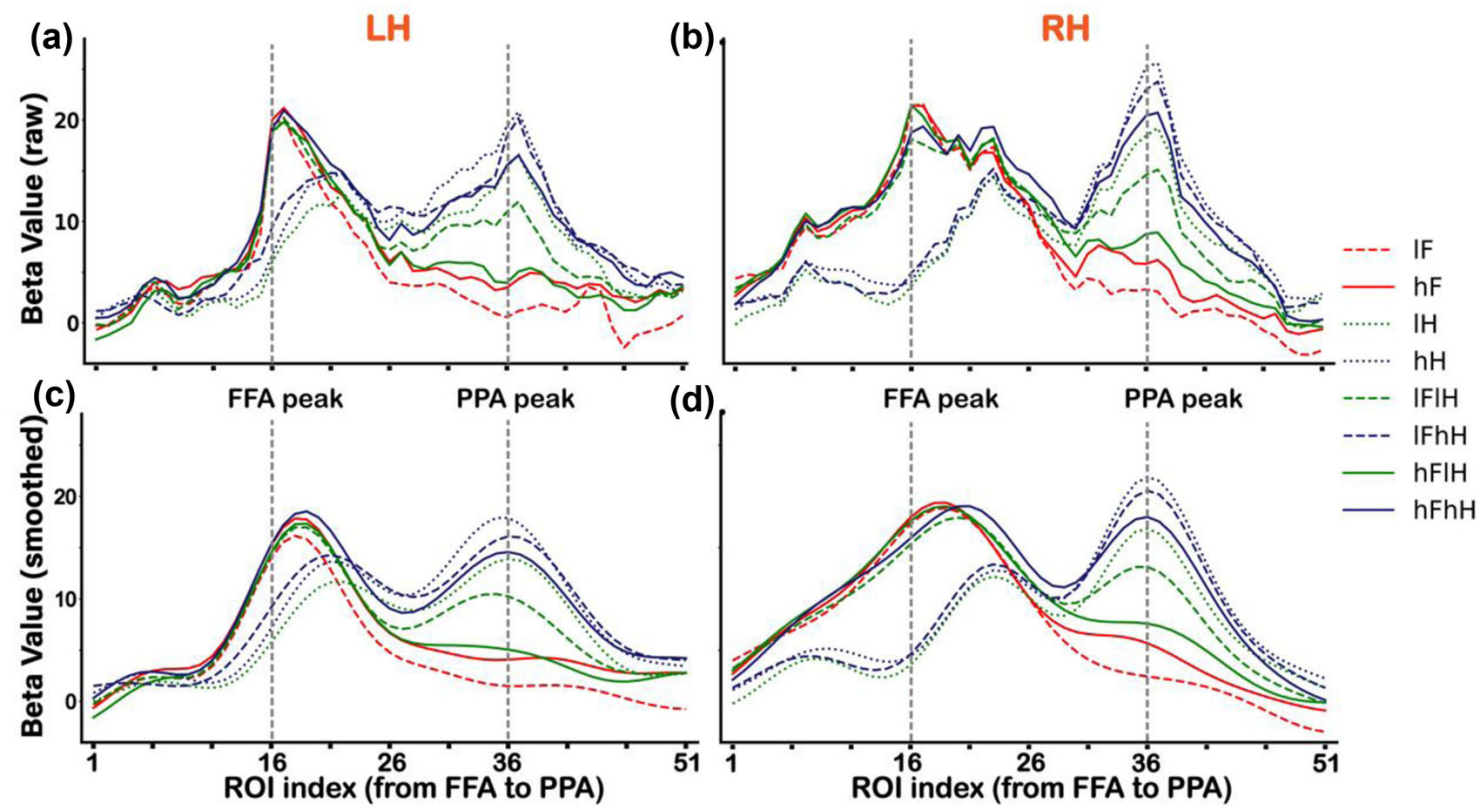
BOLD responses to all conditions (lF, hF, lH, hH, lFlH, lFhH, hFlH, and hFhH) across ROIs along the virtual line from the FFA to PPA in the main experiment. (a and b) Changes of averaged beta values to all conditions for the left (a) and right (b) hemispheres. (c and d) Smoothed beta values to all conditions for the left (c) and right (d) hemispheres. Vertical dashed lines indicate the FFA peak foci (ROI index 16) and PPA peak foci (ROI index 36). LH is the abbreviation for the left hemisphere and RH is the abbreviation for the right hemisphere

**FIGURE 3:**
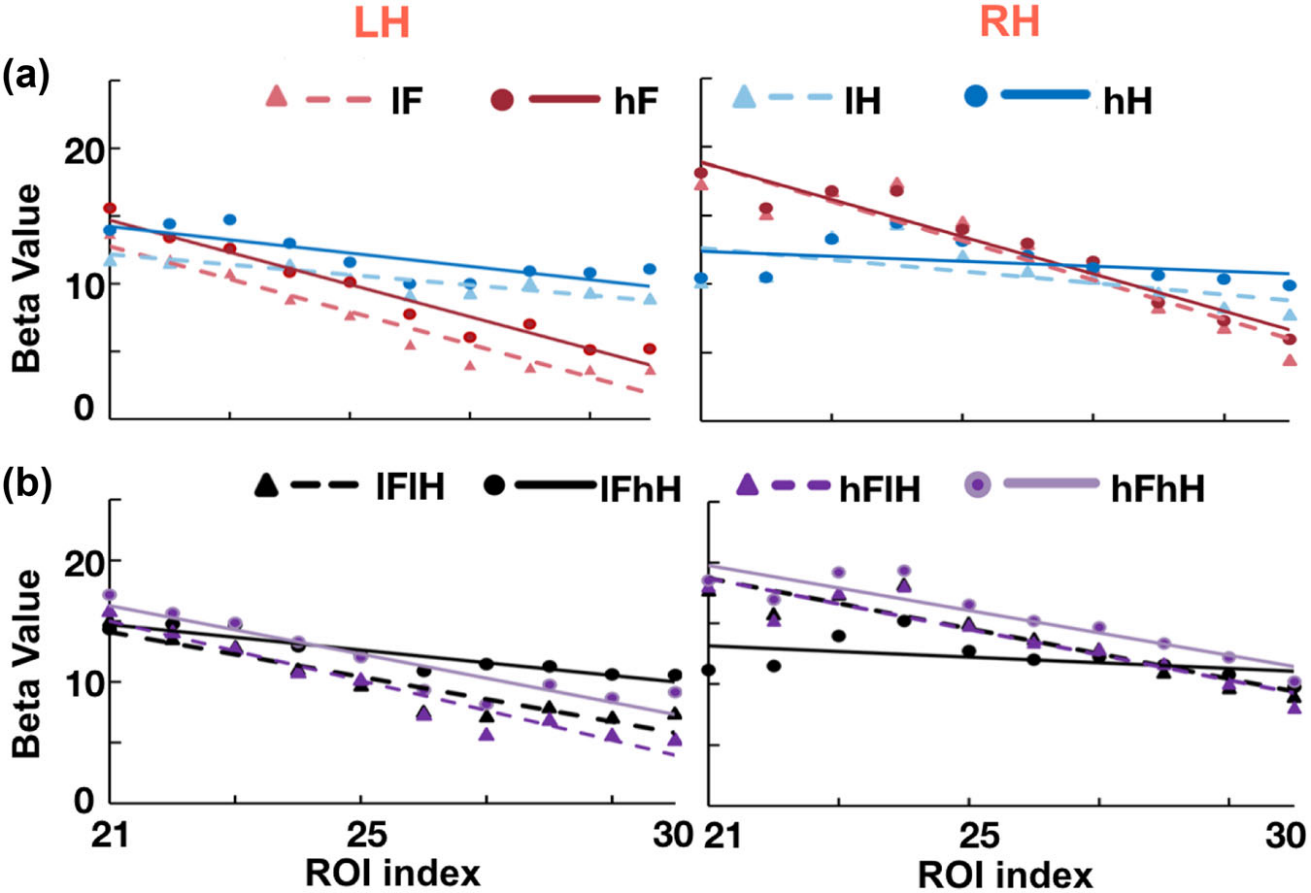
Linear regressions of beta values from ROI index 21–30 for all conditions. (a) Linear regressions of beta values for single‐image conditions (i.e., lF, lH, hF, hH). (b) Linear regressions of beta values for overlapping‐image conditions (i.e., lFlH, hFlH, hFhH, lFhH). The horizontal axes represent the ROI index from 21 to 30 along the virtual line from the FFA to PPA and the vertical axes represent the beta values for each condition. LH is the abbreviation for the left hemisphere and RH is the abbreviation for the right hemisphere

We examined the changes of BOLD responses (Figures 1(c) and 2) and selectivity (Figure 3) for faces and houses across ROIs from the FFA to PPA. For each ROI along the virtual line, we obtained the beta values by averaging 5 vertices responses for each condition (Figure 1(c) and 2). A beta value was a coefficient of the GLM (per vertex) that estimates the strength of the relationship between a covariate (e.g., stimulus condition) and BOLD signal time course. For each ROI, the averaged beta values were computed for each experimental condition across subjects and were used in subsequent statistical analyses. Selectivity was calculated separately for each subject using the formula:

Selectivity = Preferred beta values – nonpreferred beta values

For face selectivity, preferred beta values were responses to conditions containing face (i.e., lF, hF, lFlH, lFhH, hFlH, hFhH), and nonpreferred beta values represented responses to house‐only conditions (i.e., lH, hH). The selectivity for face was obtained by contrasting responses of lF versus lH, hF versus hH, lFlH versus lH, lFhH versus hH, hFlH versus lH and hFhH versus hH. For house selectivity, preferred beta values were responses to conditions containing houses (i.e., lH, hH, lFlH, lFhH, hFlH, hFhH), and nonpreferred beta values represented responses to face‐only conditions (i.e., lF, hF). The selectivity for house was obtained by contrasting responses of lH versus lF, hH versus hF, lFlH versus lF, lFhH versus lF, hFlH versus hF, and hFhH versus hF.

#### 2.5.5 Multivariate pattern analysis

Multivariate pattern analysis (MVPA) was performed using skit‐learn packages (Pedregosa et al., 2011). For each subject, a linear support vector machines (SVMs) classifier with fivefold cross‐validation was used to classify representation patterns of faces and houses. In the localizer experiment, beta values of each ROI for faces and houses were submitted to the SVM and the classification accuracies were computed by averaging output scores from fivefold cross‐validation. In the main experiment, we used a fivefold cross validation scheme for the dataset of single‐image conditions (i.e., lF, hF, lH, and hH). For each fold, we used 80% data to train a classifier and tested the classifier with the remaining 20% data. The classification accuracy for each single‐image condition was the average of the fivefold cross validation accuracies. Also, we tested the classifier with the dataset of all overlapping‐image conditions (i.e., lFlH, lFhH, hFlH, and hFhH). For each subject, the face classification accuracy was the percentage of the face category predicted form lF, hF, lFlH, lFhH, hFlH, and hFhH conditions by the SVM model in each ROI. These classification accuracies were then submitted to a two‐way repeated‐measures ANOVA (two levels of face contrast (low (lF, lFlH, and lFhH) versus high (hF, hFlH and hFhH)) × three levels of house contrast (none (lF and hF) versus low (lFlH and hFlH) versus high (lFhH and hFhH)), the solid red box in Figure 1(b)). Similarly, the house classification accuracy was the percentage of the house category predicted form lH, hH, lFlH, lFhH, hFlH, and hFhH conditions by the SVM model in each ROI. These classification accuracies were submitted to another two‐way repeated‐measures ANOVA (two levels of house contrast (low (lH, lFlH and hFlH) versus high (hH, lFhH and hFhH)) × three levels of face contrast (none (lH and hH) versus low (lFlH and lFhH) versus high (hFlH and hFhH)), the dashed blue box in Figure 1(b)).

## 3 RESULTS

### 3.1 Category preference from the FFA to PPA in the localizer experiment

Figure 1(c) shows the category preference for each ROI along the virtual line from the FFA to PPA in the localizer experiment. We can tell that the defined FFA responded larger to faces than to houses, and the defined PPA responded larger to houses than to faces. In order to statistically examine the changes of category selectivity from the FFA to PPA, we performed a paired *t*‐test between responses to faces and responses to houses for each ROI. We found significant differences in ROIs around the FFA peak (ROI index 16) and the PPA peak (ROI index 36). The significant ROIs were ROI index 16–18 in the left hemisphere and ROI index 14–19 in the right hemisphere on the FFA side, displaying a narrow distribution of face selectivity, whereas the significant ROIs were ROI index 26–43 in the left hemisphere and ROI index 33–46 in the right hemisphere on the PPA side, displaying a wide distribution of house selectivity. Furthermore, we conducted a MVPA to investigate the category representation patterns for faces versus houses across ROIs from the FFA to PPA. Figure 1(d) shows the classification accuracy for discriminating faces and houses in ROIs along the virtual line. Then, we performed a one sample t‐test between the classification accuracy and chance level (0.5) for each ROI. Significant above‐chance level ROIs were marked with stars (*ps* < .001 corrected, marked with * in Figure 1(d)).

### 3.2 Distinct category selectivity from the FFA to PPA in the main experiment

In the main experiment, Figure 2 shows BOLD responses (averaged beta values) to all conditions across ROIs from the FFA to PPA, displaying narrower responses graphs on the FFA side to faces than those on the PPA side to houses (which even appears to be a double peak). We first performed a one sample *t*‐test for BOLD responses to each condition. The significant *p* values are provided in Table S1. Then, to test the significance of face selectivity, we performed a two‐way repeated‐measures ANOVA (two levels of face contrast (low vs. high) × three levels of house contrast (none vs. low vs. high), the solid red box in Figure 1(b)) for responses to faces of each ROI from the FFA to PPA. We found significant main effects of face contrast, house contrast and an interaction effect in several ROIs (*p*s < .01, corrected). Bonferroni post hoc tests revealed that only activity of the lFhH condition (e.g., see Figure 1(b) for a demonstration) was significantly different from other conditions (*p*s < .05, corrected), which might due to the low visibility of superimposed face image in this condition. The significant *p* values are provided in Table S2. In addition, to clearly visualize the distinct patterns of category selectivity between the FFA to faces and PPA to houses, we smoothed the raw beta values by using a Gaussian process (http://github.com/SheffieldML/GPy) for each individual subject. Then, we averaged the smoothed beta values across subjects for each condition (Figures 2(c) and 2(d)).

To test the significance of house selectivity, we also performed another two‐way repeated‐measures ANOVA (two levels of house contrast (low vs. high) × three levels of face contrast (none vs. low vs. high), the dashed blue box in Figure 1(b)) for responses to houses of each ROI from the FFA to PPA. We found significant main effects of house contrast, face contrast and an interaction effect in several ROIs (*p*s < .05, corrected). Bonferroni post hoc tests revealed that the contrast of houses and overlapping presented faces could influence the activities of ROIs on the PPA side (*p*s < .05, corrected). Activities of the hFlH condition were significantly different from other conditions in several ROIs (*p*s < .05, corrected), similar to the effect of the lFhH condition. The differences between responses of ROIs on the PPA side to houses and those on the FFA side to faces were mainly reflected in the following two ways. First, there was a significant contrast effect in ROIs on the PPA side rather than on the FFA side, that is, activities of the high‐contrast house (hH) condition were significantly greater than those of the low‐contrast house (lH) condition in several ROIs around the PPA peak. Second, activities of the lH condition and lFhH condition were significantly greater than those of the lFlH condition in ROIs on the PPA side, indicating that responses of PPA to low‐contrast houses were modulated by the overlapping presented low‐contrast face. However, responses of PPA to high‐contrast houses cannot be influenced by the overlapping presented face. The significant *p* values are provided in Table S3.

It seemed that results shown in Figure 2 support our hypothesis to find asymmetrical changes in face selectiveness versus house selectiveness across the boundary between the FFA and PPA. To quantify the changes of category‐selectiveness across the boundary, we conducted linear regressions between beta values for each condition and ROIs from index 21 to 30 along the virtual line from the FFA to PPA. If the changes in face selectiveness were symmetric with those in house selectiveness, we should have seen responses to faces decrease (negative slop coefficients) and responses to houses increase (positive slop coefficients), with an “x”‐shaped pattern across the boundary. Figure 3 shows the results of linear regressions for all conditions (i.e., lF, hF, lH, hH, lFlH, lFhH, hFlH, and hFhH). We found that the regression slope coefficients were negative for all conditions (i.e., a trend of decreasing responses across the boundary), rather than an “x”‐shaped pattern. The slope coefficients for the face‐only (lF, hF) conditions were significant different from those for the house‐only (lH, hH) conditions (*p*s < .01, corrected), and the regression lines of house‐only conditions were almost flat in the horizontal direction. Statistical significance levels were shown in Table S4.

In addition, in order to evaluate any possible confounding effects of size difference between the face‐selective area and the house‐selective area, we conducted a two‐way ANOVA on the slope coefficients of responses to face‐only and house‐only conditions across ROIs on the boundary side and nonboundary side. If the differences of slopes could have been explained simply by the size difference between category selective areas, the slope coefficients of face‐only conditions on the boundary side (ROI index 1–10) should be different from those of house‐only conditions on the boundary side (ROI index 21–30) and nonboundary side (ROI index 41–50). We found significant main effects of the boundary side and interaction effect. Further paired *t*‐tests revealed a significant difference in slopes between the responses to face‐only conditions in the boundary side (ROI index 21–30) and the nonboundary side (ROI index 1–10, *p* = .001 for LH and *p* = .003 for RH, corrected). However, there were no significant difference in slopes between the responses to face‐only conditions in the boundary side (ROI index 21–30) and the responses to house‐only conditions in the nonboundary side (ROI index 41–50, *p* = .2 for LH and *p* = .6 for RH, corrected). We also found no significant differences in slops between the responses to face‐only conditions in the nonboundary side (ROI index 1–10) and the responses to house‐only conditions in the boundary side (ROI index 21–30). That is, the differences between the responses of FFA to faces and those of the PPA to house across the boundary can hardly be explained by the size differences between the face‐selective area and the house‐selective area. These results convergently suggested that distinct category tuning profiles between the FFA and PPA were mainly due to the heterogeneity of these two regions. We attached a table in supplementary materials regarding the slope coefficients for each subject (Table S14).

**FIGURE 4:**
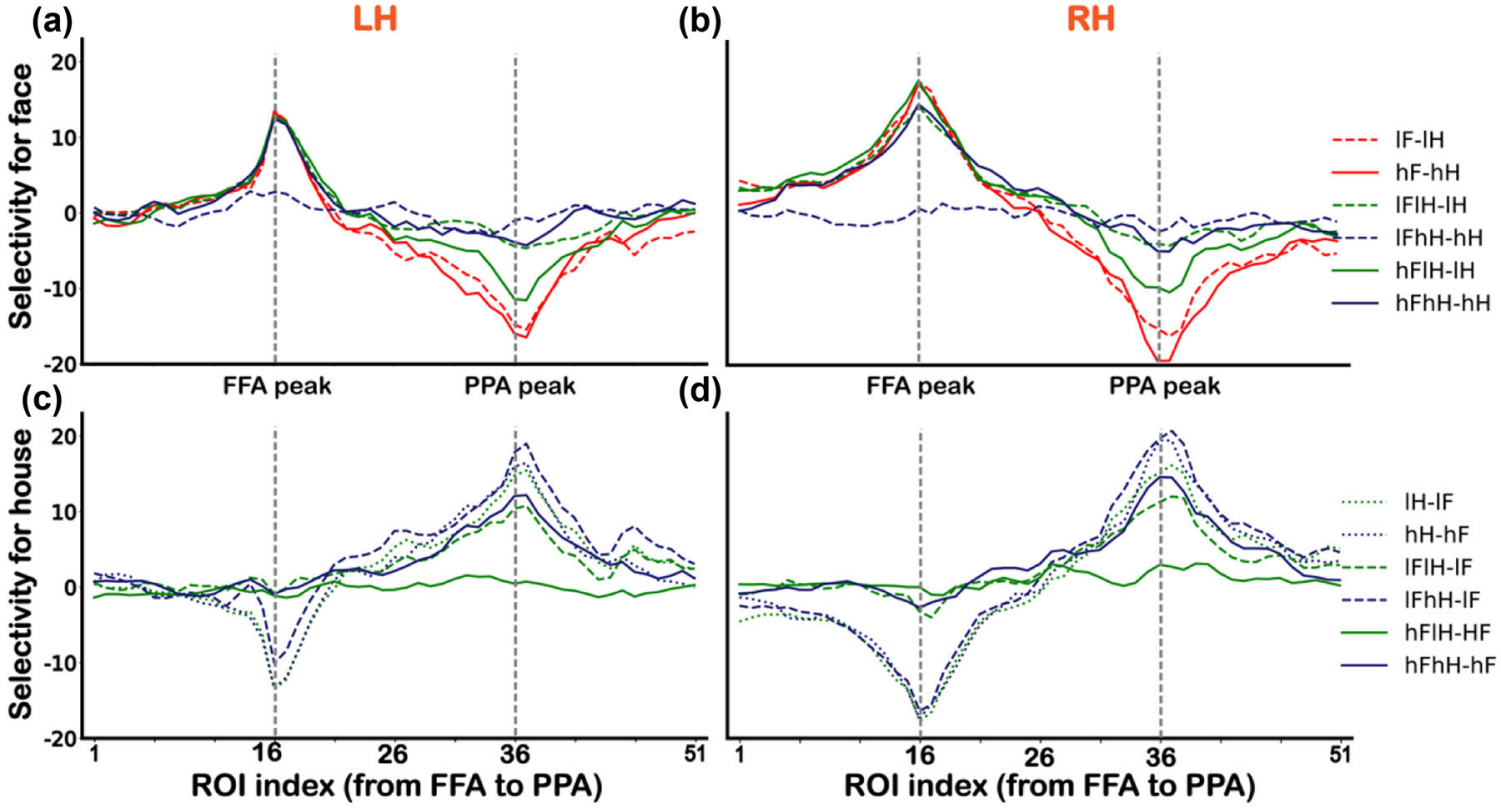
Selectivity for faces and houses in ROIs along the virtual line from the FFA to PPA. (a and b) Selectivity for the preferred face category for the left (a) and right (B) hemispheres. (c and d) Selectivity for the preferred house category for the left (c) and right (d) hemispheres. Vertical dashed lines represent the FFA peak foci (ROI index 16) and PPA peak foci (ROI index 36). LH is the abbreviation for the left hemisphere, and RH is the abbreviation for the right hemisphere

To further quantify the differences of selectivity between FFA to faces and PPA to houses, we calculated the selectivity for faces relative to houses and the selectivity for houses relative to faces (see Section 2). We found that the selectivity of ROIs on the FFA side to all conditions were only in two modes, corresponding to the difference between the presence (or high visibility) and absence (or low visibility) of a face (Figure 4). In contrast, the selectivity of ROIs on the PPA side varied more widely for all conditions, which was consistent with the results shown in Figure 2. Notably, activities of ROIs on the PPA side were modulated not only by the contrast of houses but also by the overlapping presented face, indicating relatively low selectivity. Statistical significance levels were shown in Table S5 for face selectivity and Table S6 for house selectivity.

### 3.3 Results of multivariate pattern classification accuracies from the FFA to PPA in the main experiment

**FIGURE 5:**
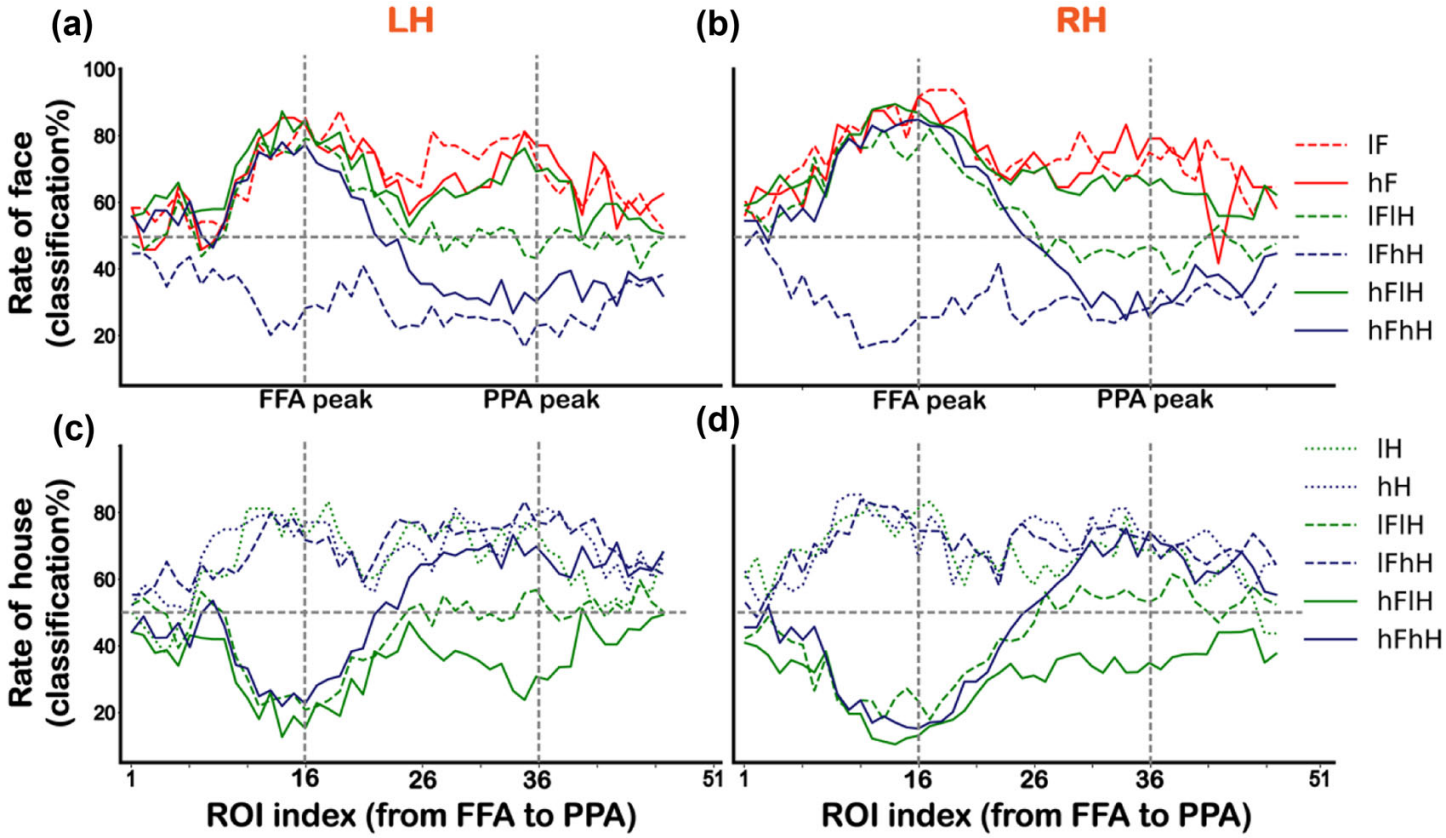
Results of decoding classification accuracies across ROIs along the virtual line from the FFA to PPA in the main experiment. (a and b) The changes of face classification accuracies corresponding to six conditions (lF, hF, lFlH, lFhH, hFlH, and hFhH) for the left (a) and right (b) hemispheres. (c and d) The changes of house classification accuracies corresponding to six conditions (lH, hH, lFlH, lFhH, hFlH, and hFhH) for the left (c) and right (d) hemispheres. Vertical dashed lines indicate the FFA peak foci (ROI index 16) and the PPA peak foci (ROI index 36). LH is the abbreviation for the left hemisphere, and RH is the abbreviation for the right hemisphere

Figure 5 shows the changes of classification accuracies for faces and houses across ROIs from the FFA to PPA. We employed a centered moving average with a 5‐length window size to smooth the classification accuracies. In this case, two start points and two end points on the virtual line were not included. A two‐way repeated‐measures ANOVA (two levels of face contrast (low vs. high) × three levels of house contrast (none vs. low vs. high), the solid red box in Figure 1(b)) was performed on face classification accuracies for each ROI along the virtual line from the FFA to PPA. The main effect of face contrast, house contrast and the interaction effect were significant in several ROIs (*p*s < .05, corrected) in both hemispheres. We further conducted a Bonferroni multiple comparison test to investigate the interaction effect. The face classification accuracy in the lFhH condition was significantly different from other conditions (*p*s < .05, corrected) in several ROIs, presumably due to low visibility of the superimposed face image in this condition, which is consistent with the univariate results. The significant *p* values are provided in Table S7.

Another two‐way repeated‐measures ANOVA (two levels of house contrast (low vs. high) × three levels of face contrast (none vs. low vs. high), the dashed blue box in Figure 1(b)) was performed on house classification accuracies for each ROI from the FFA to PPA. The main effect of house contrast, face contrast, and the interaction effect were significant in several ROIs (*p*s < .05, corrected) in both hemispheres. Multiple comparison test revealed that the house classification accuracy in the hFlH condition was significantly different from other conditions (*p*s < .05, corrected) in several ROIs, similar to the effect of the lFhH condition described above. Consistent with a previous report (Guo & Meng, 2015), no difference was found between the low‐contrast house (lH) condition and the high‐contrast house (hH) condition, indicating that the contrast alone did not affect the representation pattern of houses in ROIs on the PPA side. However, the house classification accuracies in the lFhH condition and lH condition were significantly larger than those in the lFlH condition, suggesting that the representation pattern of house was affected by the overlapping presented low‐contrast face. The significant *p* values are provided in Table S8.

In addition, we adopted a surface‐based searchlight decoding analysis to assess local regions whose activity patterns distinguish between face and house categories throughout the whole brain. Given that the FFA and PPA were defined from the activation‐based GLM analysis by contrasting face and house conditions in the localizer scan, the searchlight analysis maximizes the possibility of finding vertices outside of the defined FFA and PPA that may be preferentially associated with the perception of faces and houses (Chen et al., 2011). This analysis was performed in the native space of individual subject by using Nilearn and scikit‐learn packages (Abranham et al., 2014; https://github.com/nilearn/nilearn). We first defined an adjacency matrix based on the inflated gray matter surface mesh such that nearby vertices were concatenated within the same searchlight. Then, we employed a 5 mm radius of disks to construct a searchlight structure for each vertex of the surface mesh. We used the dataset of face‐only (i.e., lF, hF) and house‐only (i.e., lH, hH) conditions in the main experiment to train and test a linear Ridge regression classifier, with all parameters set to default values as provided by the scikit‐learn packages (alpha = 10). For each vertex, a fivefold cross‐validation classifier was performed to distinguish between faces and houses. This procedure yielded an information‐based map highlighted regions containing category information on the cortical surface. For each subject, the searchlight information‐based map showed considerable overlap with the activation‐based map from the univariate GLM analysis of the localizer data. To assess the statistical validity of the decoding accuracy map on the group level, we transformed the accuracy map of individual subject into the standard surface space (fsaverage). Then, a one sample *t*‐test was performed to compare the classification accuracy against the chance level (0.5) for each vertex. Figure S3 shows the results of group analysis of searchlight decoding accuracy. Colored vertices indicate searchlight clusters with significantly above‐chance classification accuracy (chance level = 0.5, *p* < .001, uncorrected). These results, together with our ROI analyses, suggested that the defined FFA and PPA were highly sensitive and selective to faces and houses, respectively.

### 3.4 Results of the main experiment from the occipital pole to the FFA and from the occipital pole to the PPA

**FIGURE 6:**
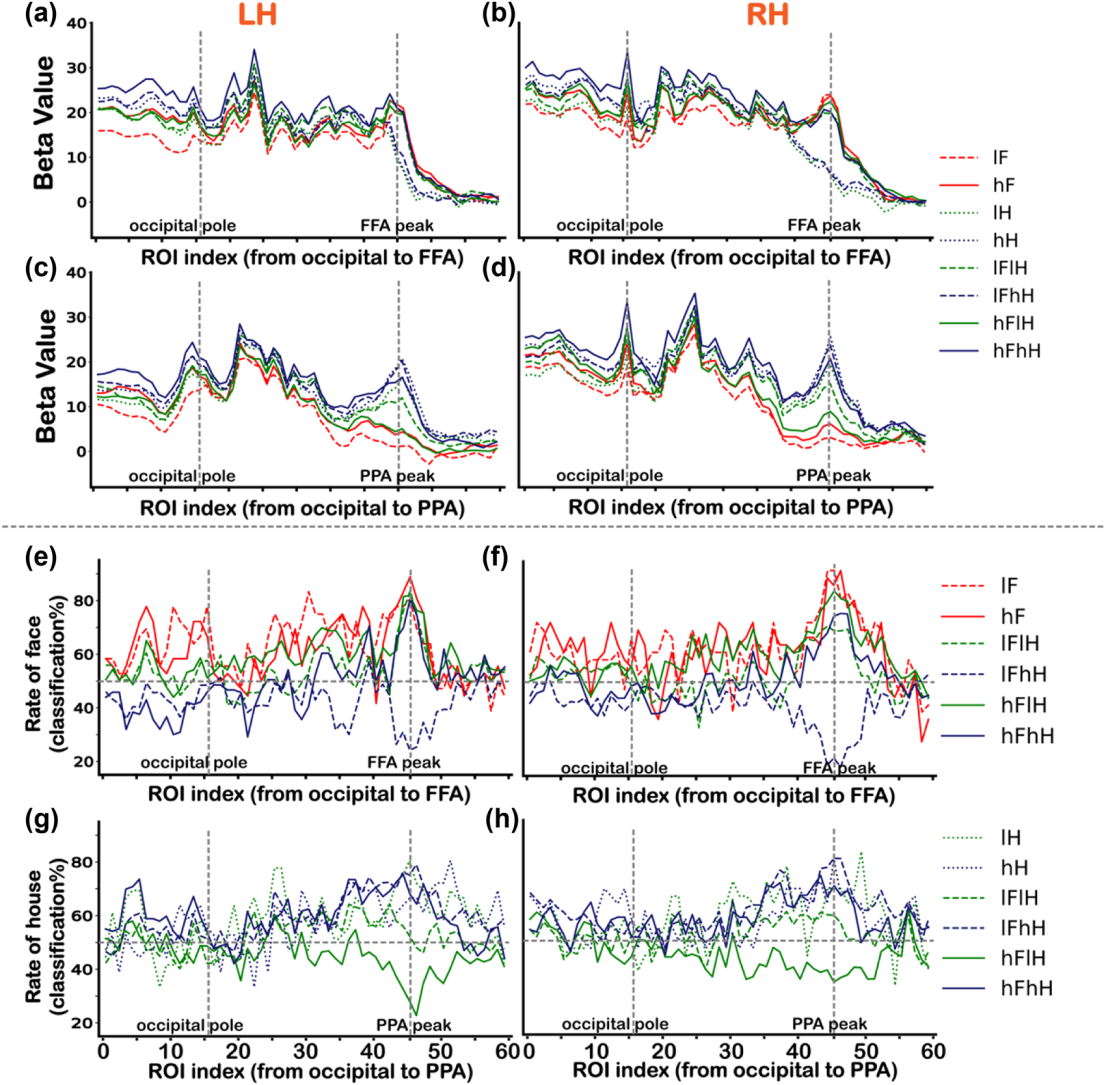
Univariate and multivariate results of the main experiment from the occipital pole to the FFA/PPA. (a and b) The changes of BOLD activities to all conditions across ROIs along the virtual line from the occipital pole to the FFA for the left (a) and right (b) hemispheres. (c and d) The changes of BOLD activities to all conditions across ROIs along the virtual line from the occipital pole to the PPA for the left (c) and right (d) hemispheres. (e and f) The changes of face classification accuracies corresponding to six conditions (lF, hF, lFlH, lFhH, hFlH, and hFhH) across ROIs along the virtual line from the occipital pole to the FFA for the left (e) and right (f) hemispheres. (g and h) The changes of house classification accuracies corresponding to six conditions (lH, hH, lFlH, lFhH, hFlH, and hFhH) across ROIs along the virtual line from the occipital pole to the PPA for the left (g) and right (h) hemispheres. Vertical dashed lines represent the occipital pole (ROI index 16) and the FFA/PPA peak foci (ROI index 46). LH is the abbreviation of the left hemisphere, and RH is the abbreviation of the right hemisphere

To further confirm the differences of category selectivity were specific to the category selective regions, we investigated the changes of BOLD activities and classification accuracies across ROIs from the occipital pole to the FFA and PPA, respectively. Consistent with the results shown in Figure 2, for all conditions, the responses varied more widely around the PPA to houses than those around the FFA to faces (Figures 6(a)–6(d)). For each ROI along the virtual line from the occipital pole to the FFA, we conducted a two‐way ANOVA (two levels of face contrast (low vs. high) × three levels of house contrast (none vs. low vs. high), the solid red box in Figure 1(b)) for responses to faces. We found statistically significant main effects and an interaction effect in several ROIs (*p*s < .05, corrected) in both hemispheres. Multiple comparisons revealed that the contrast effect was in the occipital cortex not in the FFA, except for the low visibility of overlapping presented face condition (lFhH). The significant *p* values are displayed in Table S9. For each ROI along the virtual line from the occipital pole to the PPA, another two‐way ANOVA (two levels of house contrast (low vs. high) × three levels of face contrast (none vs. low vs. high), the dashed blue box in Figure 1(b)) was performed for responses to houses. We found significant main effects and an interaction effect in several ROIs (*p*s < .05, corrected). Multiple comparisons revealed that activity of the hH condition was significantly greater than that of the lH condition in several ROIs along the virtual line. Activity of the lFhH condition was significantly greater than that of the lFlH condition in ROIs around the PPA peak, which was consistent with results shown in Figure 2. The significant *p* values are displayed in Table S10.

Figures 6(e) and 6(f) show the changes of face classification accuracies across ROIs from the occipital pole to the FFA. Figures 6(g) and 6(h) show the changes of house classification accuracies across ROIs from the occipital pole to the PPA. Two‐way ANOVA revealed a significant interaction effect in several ROIs (*p*s < .05, corrected). Multiple comparisons revealed that significant effects of contrast were in the occipital cortex not in the FFA or PPA, except for the low visibility of overlapping presented face condition (lFhH) and low visibility of overlapping presented house condition (hFlH). The significant *p* values are displayed in Table S11 and S12. These results, together with univariate and multivariate results across ROIs from the FFA to PPA, suggested a better face selectiveness in the FFA than house selectiveness in the PPA. To graphically display the distinct response properties between the FFA and PPA, we also attached results of the functional localizer scan along virtual lines from the occipital pole to the FFA and PPA in Figure S2.

## 4 DISCUSSION

The main finding of this study is that response properties of the FFA to faces are different from those of the PPA to houses. First, responses of the FFA were invariant to the changes in low‐level visual features (i.e., contrast), while responses of the PPA were modulated by stimulus contrast. Second, responses of the FFA were highly selective to faces and invariant to the concurrently presented house image, whereas responses of the PPA were significantly susceptible to the concurrently presented face image. Third, responses of ROIs to faces across the presumed boundary from face‐selective to house‐selective decreased sharply, suggesting a boundary line of face‐selective area. In contrast, responses of ROIs to houses across the presumed boundary from house‐selective to face‐selective only slightly decreased and surprisingly increased again on the FFA side, making it difficult to determine a borderline of house‐selective area. By conducting linear regressions and comparing the slopes of response changes, we found asymmetry changes of response properties from the FFA to PPA for faces versus from the PPA to FFA for houses. Further analyses of multivariate representation patterns demonstrated that the representation of preferred stimulus was more robust in the FFA than in the PPA, confirming that underlying mechanism of face processing in the FFA is different from house processing in the PPA.

Responses of the PPA were modulated by low‐level features, such as image contrasts and concurrently‐presented images. However, distinct response properties between the FFA to faces and the PPA to houses can hardly be explained by any effects of difference in low‐level visual features for the following reasons. First, stimulus contrast was carefully controlled by using the SHINNE toolbox. Second, by analyzing multivariate representation patterns in ROIs from the occipital pole to the FFA and PPA, we did not observe any significant differences of classification accuracy between face category and house category in the occipital lobe, both in the main and localizer experiment data. Third, consistent with our results, low‐level visual features (such as, spatial frequency, orientation, contrast) modulated responses in the house‐selective region have been reported in previous studies (Berman et al., 2017; Epstein & Baker, 2019; Groen et al., 2017; Guo & Meng, 2015; Nasr et al., 2014; Nasr & Tootell, 2012; Rajimehr et al., 2011; Watson et al., 2014). High‐level aspects of house category information and low‐level visual features are inextricably linked in the PPA, indicating the complex nature of processing within the PPA.

Why do the FFA and PPA display distinct response properties? A revised normalization model was proposed to account for different response profiles of category‐selective areas to variations of low‐level visual features (e.g., contrast, size) and concurrently presented multiple stimuli (Bao & Tsao, 2018; Kliger & Yovel, 2020). The framework of revised normalization model refers to an operation in which responses of neurons are divided by a common factor representing summed activities of a pool of neighboring neurons (Carandini & Heeger, 2012). That is, responses of a given ROI are determined not only by the presented stimulus but also by the homogeneity of neighboring neurons (Baeck et al., 2013; MacEvoy & Epstein, 2009; Zoccolan et al., 2007). If the category selectivity of neighboring areas of a given ROI is homogenous, the normalization pool should be unresponsive to nonpreferred stimulus and the normalization factor would be very small. Therefore, this ROI would be highly selective to the preferred stimuli and invariant to changes in low‐level features as well as concurrently presented nonpreferred stimulus, coinciding with the response property of the FFA to faces. However, if the category selectivity of neighboring areas of a given ROI is heterogenous, the normalization pool would respond to presented stimulus to a large extent. Thus, responses of the given ROI to the preferred stimuli would be normalized by neighboring areas, which is similar to the response property of the PPA to houses. For our results, responses of the PPA to houses might be normalized according to low‐level visual features of the presented stimuli (i.e., contrast) and a concurrently presented face.

Consistent with our results, differences between face neurons/areas and other category‐selective neurons/areas have been reported by several studies (Bell et al., 2011; Tsao et al., 2006). These findings, together with ours, support the notion that faces may be a special class of visual stimuli and face neurons may be a special type in temporal cortex. Many perceptual studies have shown that human faces attract and modulate attention more quickly and reliably than other object categories (Hershler & Hochstein, 2005; Morrisey et al., 2019; Ro et al., 2007). It is not new that faces have been thought to be processed by a specialized mechanism, such as holistic processing (Maurer et al., 2002; Richler & Gauthier, 2014) and a special configuration that can be disrupted by inversion (Pallett & Meng, 2015). Moreover, the priority of face processing may be innate in humans, for instance, newborns tended to look longer normal faces than scrambled faces (Morton & Johnson, 1991; Cassia et al., 2004 Turati et al., 2008; Tsao & Livingstone, 2008). As the specific mechanism of face processing, the decrease in the rate of house classification may not necessarily be only owing to the specific attention‐grabbing power of faces. In the present study, there were no differences between the hH and lFhH conditions both in univariate vertex‐wise activities and multivariate pattern results. The rate of house classification is hurt by the presence of faces only when the concurrently presented house image was in the low contrast (i.e., lH vs. lFlH), suggesting that the response property of PPA was modulated by the attributes of house images.

Our study is the first to directly compare the response properties of two neighboring category‐selective areas on the inflated relatively extensive cortical surface. Nonetheless, recent studies examined the within‐category organization of neural representations (e.g., face parts and face features) on a fine‐scale by using ultra‐high field fMRI and vertex‐wise tuning models (de Haas et al., 2021; Zhang et al., 2021). In addition, while we focused on a virtual line connecting the FFA and PPA, which often runs along the medial‐lateral axis of human ventral temporal cortex, there are putative functional differences along the posterior‐anterior axis of ventral temporal cortex (Silson et al., 2019; Steel et al., 2021; Weiner et al., 2017). Future studies may combine our approach and ultra‐high field fMRI, focusing on population tuning functions of neurons near the boundary of category specific regions, to further study how human ventral temporal cortex is partitioned and organized on a fine‐scale along the posterior‐anterior axis.

In general, there have been several models of how object representations are organized in the ventral visual pathway. Most notably, ventral temporal cortex is composed of discrete patches specializing in individual visual category (Reddy & Kanwisher, 2006). The opposite hypothesis propose that objects are coded via a distributed neural system across the ventral visual cortex (Haxby, et al., 2001; Tanaka et al., 1991). However, ample behavioral and neuroimaging evidence indicated that neither the modular organization nor the distributed model alone could account for the complicated neural machinery of object representation. For this reason, a hybrid modular‐distributed organization model of object representation was proposed, suggesting that objects were represented by a series of highly dedicated and distributed category‐selective clusters, and that weakly selective or nonselective voxels outside of these clusters were also involved in neural coding (Cohen & Tong, 2001; O'Toole et al., 2005; Shehzad & McCarthy, 2018; Weiner & Grill‐Spector, 2010). Our data, on the one hand, confirmed the highly selective and invariant response properties of the FFA, indicating that neural representations of faces utilized a dedicated cortical area. On the other hand, responses of ROIs on the PPA side were modulated not only by low‐level visual features (i.e., contrast) but also by the concurrently presented face image, suggesting that face neurons may also existed outside the face‐selective area, in line with a more distributed organizational scheme. Taken together, our results suggest that potentially different computations were performed by distinct category‐selective clusters, supporting the hybrid organization model, which incorporated both dedicated modules and relatively distributed elements (Bell et al., 2011; Cohen & Tong, 2001; Kriegeskorte et al., 2008; O'Toole et al., 2005; Shehzad & McCarthy, 2018; Weiner & Grill‐Spector, 2010).

## 5 CONCLUSION

Compared with highly selective and invariant response properties of the FFA, activities of the PPA are more susceptible to changes in low‐level visual features (i.e., contrast) and a concurrently presented stimuli (i.e., faces). In addition, response properties of ROIs across the boundary between the FFA and PPA are asymmetrical from face‐selective to house‐selective relative to from house‐selective to face‐selective. These results convergently suggest distinct response properties between the FFA to faces and the PPA to houses, supporting a combination of specialized modules and relatively distributed organization of object representation in ventral temporal cortex (Bell et al., 2011; Cohen & Tong, 2001; O'Toole et al., 2005; Weiner & Grill‐Spector, 2010). Consequently, theories of object representation mapping in category‐selective areas should perhaps consider a series of dedicated category‐selective clusters and minimally overlapping distributed systems.

## CONFLICT OF INTEREST

The authors declare no potential conflict of interest.

### PEER REVIEW

The peer review history for this article is available at https://publons.com/publon/10.1002/brb3.2706

## Supporting information

Supplementary Tables

## Data Availability

All data will be available upon publication to the neuroimaging research community in the public domain. This data sharing complies with the requirements of South China Normal University and the National Natural Science Foundation of China.
