# Supplementary material for: Distinct response properties between the FFA to faces and the PPA to houses
Source: Brain Behav. 2022 Jul 18;12(8):e2706. doi: 10.1002/brb3.2706 (PMC9392545; doi:10.1002/brb3.2706)

Supplementary Materials

Table 1: Results of one sample t-test for the averaged beta values under all conditions: lF, hF, lH, hH, lFlH, lFhH, hFlH, and hFhH in ROIs along the virtual line from the FFA to PPA in the main experiment (Figure 2). 0.001 indicates the *p* value is less than 0.001. LH is the abbreviation for the left hemisphere and RH is the abbreviation for the right hemisphere.

***p* values**	**LH**								**RH**							
	**One sample ttest**								**One sample ttest**							
**ROI index**	**hF**	**hFhH**	**hFlH**	**hH**	**lF**	**lFhH**	**lFlH**	**lH**	**hF**	**hFhH**	**hFlH**	**hH**	**lF**	**lFhH**	**lFlH**	**lH**
**1**															0.008	
**2**									0.009						0.007	
**3**													0.007			
**4**													0.005		0.009	
**5**													0.005			
**6**																
**7**														0.01		
**8**									0.002	0.002	0.001	0.002	0.003	0.002	0.002	
**9**	0.01								0.001	0.001	0.001	0.001	0.001		0.001	
**10**									0.001	0.002	0.001	0.003	0.001		0.002	
**11**					0.01				0.001	0.002	0.001	0.007	0.001		0.002	
**12**			0.008		0.002				0.001	0.001	0.001		0.001		0.002	
**13**					0.002				0.001	0.001	0.001		0.001		0.001	
**14**	0.006	0.003							0.001	0.001	0.001	0.006	0.001		0.001	
**15**	0.001	0.001	0.001		0.001		0.001		0.001	0.001	0.001		0.001		0.001	
**16**	0.001	0.001	0.001		0.001	0.009	0.001		0.001	0.001	0.001		0.001		0.001	
**17**	0.001	0.001	0.001		0.001	0.005	0.001		0.001	0.001	0.001		0.001		0.001	
**18**	0.001	0.001	0.001	0.008	0.001	0.004	0.001		0.001	0.001	0.001	0.007	0.001	0.003	0.001	
**19**	0.001	0.001	0.001	0.001	0.001	0.002	0.001	0.002	0.001	0.001	0.001	0.008	0.001	0.004	0.001	0.008
**20**	0.001	0.001	0.001	0.001	0.001	0.002	0.001	0.002	0.001	0.001	0.001	0.001	0.001	0.001	0.001	0.001
**21**	0.003	0.001	0.002	0.001	0.001	0.002	0.001	0.002	0.001	0.001	0.001	0.006	0.001	0.001	0.001	0.005
**22**	0.007	0.002	0.007	0.001	0.004	0.003	0.002	0.002	0.001	0.001	0.001	0.003	0.001	0.001	0.001	0.001
**23**	0.004	0.001	0.004	0.002	0.006	0.001	0.003	0.002	0.001	0.001	0.001	0.001	0.001	0.001	0.001	0.001
**24**	0.003	0.001	0.003	0.002		0.001	0.002	0.002	0.001	0.001	0.001	0.001	0.001	0.001	0.001	0.001
**25**		0.006		0.004		0.001		0.004	0.001	0.001	0.001	0.001	0.001	0.001	0.001	0.001
**26**		0.002		0.004		0.001		0.005	0.002	0.001	0.001	0.001	0.005	0.001	0.001	0.001
**27**	0.004	0.001		0.002		0.001	0.004	0.002	0.002	0.001	0.001	0.001		0.001	0.001	0.001
**28**		0.001		0.001		0.001	0.003	0.001	0.002	0.001	0.001	0.001		0.001	0.001	0.004
**29**		0.003		0.001	0.009	0.001	0.002	0.001		0.001	0.005	0.001		0.002	0.001	0.001
**30**	0.009	0.004		0.001		0.001	0.003	0.002		0.001		0.002		0.004	0.001	0.001
**31**		0.002		0.002		0.002	0.004	0.007	0.005	0.001	0.001	0.001		0.002	0.001	0.001
**32**		0.003		0.003		0.002	0.006		0.001	0.001	0.001	0.001		0.001	0.001	0.001
**33**		0.005		0.004		0.002	0.009	0.009	0.001	0.001	0.001	0.001		0.001	0.001	0.001
**34**		0.002		0.001		0.002	0.004	0.002	0.001	0.001	0.001	0.001		0.001	0.001	0.001
**35**		0.001		0.001		0.001	0.003	0.001	0.004	0.001	0.001	0.001		0.001	0.001	0.001
**36**		0.001		0.001		0.001	0.002	0.001	0.005	0.001	0.005	0.001		0.001	0.001	0.001
**37**		0.002		0.001		0.001	0.004	0.003	0.008	0.001	0.005	0.001		0.001	0.001	0.001
**38**		0.007		0.001		0.001	0.009	0.004		0.002		0.001		0.001	0.006	0.002
**39**		0.005		0.001		0.001		0.005		0.005		0.001		0.001	0.006	0.001
**40**		0.004		0.001		0.001		0.004		0.007		0.001		0.001		0.003
**41**		0.009		0.006		0.001		0.003				0.002		0.006		
**42**						0.004		0.004				0.002				
**43**												0.006				
**44**												0.003		0.005		0.009
**45**												0.005				0.009
**46**																0.01
**47**																
**48**																
**49**																
**50**																
**51**																

Table 2: Results of two-way ANOVA for responses to faces (2 levels of face contrast (low (lF, lFlH and lFhH) *vs.* high (hF, hFlH and hFhH)) * 3 levels of house contrast (none (lF and hF) *vs.* low (lFlH and hFlH) *vs.* high (lFhH and hFhH)), the solid red box in the Figure 1B) in ROIs along the virtual line from the FFA to PPA in the main experiment (Figure 2A and 2B). 0.001 indicates the *p* value is less than 0.001. LH is the abbreviation for the left hemisphere and RH is the abbreviation for the right hemisphere.

***p* values**		LH											
		Main effect of face contrast	Main effect of house contrast	Interaction effect	Multiple Comparisons								
					hFhH vs. hF	hFhH vs. hFlH	hFlH vs. hF	lFhH vs. lF	lFhH vs. lFlH	lFlH vs. lF	lFhH vs. hFhH	lF vs. hF	hFlH vs.lFlH
ROI index	1		0.016										
	2												
	3												
	4												
	5												
	6												
	7												
	8												
	9												
	10												
	11												
	12												
	13												
	14												
	15	0.023									0.038		
	16	0.001	0.002	0.001				0.005	0.004		0.001		
	17	0.001	0.024	0.001				0.023	0.01		0.001		
	18	0.002		0.007							0.004		
	19	0.015											
	20												
	21												
	22		0.04										
	23		0.027										
	24		0.012										
	25		0.005	0.021									
	26		0.001	0.001				0.006	0.012	0.038			
	27		0.001	0.004				0.001	0.012	0.009		0.033	
	28		0.001	0.011	0.035			0.001	0.001				
	29		0.001					0.003					
	30		0.001		0.043	0.047		0.009					
	31		0.001	0.03	0.006	0.012		0.015					
	32		0.001	0.011	0.012	0.024		0.008		0.035			
	33		0.001	0.005	0.021	0.04		0.008	0.021	0.046			
	34		0.001	0.001	0.004	0.007		0.003	0.022	0.007			
	35		0.001	0.001	0.004	0.001		0.001	0.008	0.001			0.011
	36		0.001	0.001	0.004	0.001		0.001	0.001	0.001			0.01
	37		0.001	0.001	0.005	0.001		0.001	0.001	0.003	0.041		0.031
	38		0.001	0.001	0.01	0.001		0.001	0.001	0.019			0.046
	39		0.001	0.006	0.017	0.001		0.001	0.002				
	40		0.001	0.012	0.009	0.002		0.001	0.003	0.027			
	41		0.001	0.016	0.012	0.001		0.001	0.005	0.027			
	42		0.001			0.002		0.001	0.012				
	43		0.008			0.002							
	44		0.012			0.009							
	45		0.001	0.019				0.005					
	46		0.002	0.005				0.006		0.024		0.044	
	47		0.01	0.015				0.04					
	48		0.011	0.013						0.031		0.018	
	49		0.021									0.022	
	50	0.012	0.035										
	51	0.024											

p values		RH											
		Main effect of face contrast	Main effect of house contrast	Interaction effect	Multiple Comparisons								
					hFhH vs. hF	hFhH vs. hFlH	hFlH vs. hF	lFhH vs. lF	lFhH vs. lFlH	lFlH vs. lF	lFhH vs. hFhH	lF vs. hF	hFlH vs.lFlH
ROI index	1												
	2												
	3												
	4												
	5			0.039									
	6												
	7												
	8			0.042									
	9	0.019	0.049	0.008							0.04		
	10	0.007	0.047	0.003							0.02		
	11	0.004	0.018	0.001				0.047	0.039		0.012		
	12	0.002	0.009	0.001				0.026	0.022		0.004		
	13	0.002	0.003	0.001				0.017	0.012		0.002		
	14	0.001	0.001	0.001				0.004	0.004		0.001		
	15	0.001	0.001	0.001				0.001	0.001		0.001		
	16	0.001	0.001	0.001				0.001	0.001		0.001		0.049
	17	0.001	0.001	0.001				0.01	0.009		0.001		
	18	0.007	0.001	0.001				0.01	0.036		0.004		
	19	0.008	0.001	0.001				0.006	0.008		0.003		
	20	0.02	0.018	0.001					0.045		0.002		
	21			0.01							0.026		
	22			0.033							0.035		
	23												
	24			0.033									
	25												
	26												
	27		0.021		0.044								
	28		0.026		0.046								
	29		0.016										
	30		0.009										
	31		0.01							0.037			
	32		0.002	0.045				0.02		0.019			
	33		0.001	0.012		0.022		0.005		0.013			
	34		0.001	0.027		0.021		0.003		0.038			
	35		0.001		0.032	0.026		0.004					
	36		0.001		0.017	0.03		0.005					
	37		0.001	0.022	0.007	0.017		0.001	0.029	0.005			
	38		0.001	0.013	0.006	0.01		0.001	0.033	0.006			
	39		0.001		0.009	0.008		0.001		0.002			
	40		0.001		0.006	0.035		0.001		0.012			
	41		0.001		0.036			0.002					
	42		0.001					0.006					
	43		0.001					0.007	0.043				
	44		0.001		0.012			0.003	0.034				
	45		0.001		0.01			0.018					
	46		0.003										
	47												
	48												
	49												
	50							0.032					
	51							0.03					

Table 3: Results of two-way ANOVA for responses to houses (2 levels of house contrast (low (lH, lFlH and hFlH) *vs.* high (hH, lFhH and hFhH)) * 3 levels of face contrast (none (lH and hH) *vs.* low (lFlH and lFhH) *vs.* high (hFlH and hFhH), the dashed blue box in the Figure 1B) in ROIs along the virtual line from the FFA to PPA in the main experiment (Figure 2A and 2B). 0.001 indicates the *p* value is less than 0.001. LH is the abbreviation for the left hemisphere and RH is the abbreviation for the right hemisphere.

***p* values**	LH											
	Main effect of face contrast	Main effect of house contrast	Interaction effect	Multiple Comparisons								
ROI index				hFhH vs. hFlH	lFhH vs. lFlH	hH vs. lH	hFhH vs. lFhH	hFhH vs. hH	lFhH vs. hH	hFlH vs. lFlH	hFlH vs. lH	lFlH vs. lH
1		0.023		0.045								
2		0.014										
3		0.015										
4		0.043										
5												
6												
7												
8												
9												
10												
11	0.043											
12	0.005											
13	0.002											0.038
14	0.002							0.003				0.017
15	0.001		0.003					0.001			0.003	0.001
16	0.001	0.031	0.001		0.002		0.001	0.001			0.001	0.001
17	0.001		0.001		0.005		0.001	0.001			0.001	0.001
18	0.001		0.001				0.008	0.003			0.004	0.001
19	0.005					0.029						0.026
20												
21												
22		0.045										
23		0.025										
24		0.017										
25		0.006			0.034							
26		0.001	0.042		0.006							
27		0.001			0.006							
28	0.048	0.001			0.001							
29	0.037	0.001		0.026	0.03							
30	0.017	0.001		0.024		0.024						
31	0.042	0.001		0.006		0.027						
32	0.039	0.002		0.012								
33	0.041	0.004		0.02	0.01							
34	0.007	0.002	0.019	0.003	0.011							
35	0.001	0.001	0.001	0.001	0.004					0.021	0.005	0.012
36	0.001	0.001	0.001	0.001	0.001					0.021	0.004	0.021
37	0.001	0.001	0.004	0.001	0.001	0.047					0.01	0.017
38	0.001	0.001	0.026	0.001	0.001	0.036					0.006	0.015
39	0.002	0.001	0.025	0.001	0.001						0.018	
40	0.006	0.001	0.032	0.001	0.002						0.024	
41		0.001	0.011	0.001	0.002						0.021	
42		0.002	0.005	0.001	0.006							
43		0.005	0.033	0.001								
44		0.009		0.004								
45		0.007		0.027								
46		0.007		0.043								
47		0.028										
48		0.034										
49		0.046										
50		0.026										
51												

***p* values**	RH											
				Multiple Comparisons								
ROI index	Main effect of face contrast	Main effect of house contrast	Interaction effect	hFhH vs. hFlH	lFhH vs. lFlH	hH vs. lH	hFhH vs. lFhH	hFhH vs. hH	lFhH vs. hH	hFlH vs. lFlH	hFlH vs. lH	lFlH vs. lH
1												
2												
3												
4												
5	0.021		0.034									
6	0.037		0.049									
7	0.031		0.041									
8	0.015		0.025									
9	0.005		0.007									
10	0.002		0.005		0.04		0.041	0.04				
11	0.001		0.001		0.019		0.024	0.019				0.041
12	0.001		0.001		0.011		0.007	0.003			0.03	0.014
13	0.001	0.032	0.001		0.006		0.005	0.001			0.005	0.002
14	0.001	0.004	0.001		0.002		0.001	0.001			0.001	0.001
15	0.001	0.001	0.001		0.001		0.001	0.001			0.001	0.001
16	0.001	0.001	0.001		0.001		0.001	0.001			0.001	0.001
17	0.001	0.024	0.001		0.005		0.003	0.001			0.001	0.001
18	0.001	0.039	0.001		0.018		0.008	0.002			0.006	0.004
19	0.001	0.006	0.001		0.004		0.006	0.016			0.016	0.007
20	0.002		0.001		0.023		0.004	0.025				
21	0.039		0.023									
22	0.05											
23												
24			0.045									
25												
26												
27												
28												
29												
30												
31												
32		0.036				0.04						
33		0.008		0.011		0.019						
34	0.007	0.002		0.01		0.014						
35	0.003	0.001		0.013	0.042	0.013						
36	0.003	0.001		0.015	0.035	0.027						
37	0.001	0.001		0.008	0.015	0.015					0.038	
38	0.003	0.001		0.005	0.016	0.009					0.031	
39	0.007	0.001		0.004		0.003					0.046	
40	0.03	0.001		0.018	0.047	0.003						
41	0.04	0.003			0.048	0.022						
42	0.04	0.005				0.04						
43	0.023	0.003			0.021							
44	0.02	0.001		0.04	0.017							
45	0.022	0.004										
46												
47												
48												
49												
50	0.01											
51	0.001										0.024	

Table 4: Slopes, intercepts and corresponding *p* values of the averaged beta values under all conditions: lF, hF, lH, hH, lFlH, lFhH, hFlH, and hFhH across ROIs from index 21 to index 30 along the virtual line from the FFA to PPA in the main experiment (Figure 3). 0.001 indicates the *p* value is less than 0.001. LH is the abbreviation for the left hemisphere and RH is the abbreviation for the right hemisphere.

	LH			RH		
	Slope	***p* values (pairwise ttest)**	***Intercept***	***Slope***	***p* values (pairwise ttest)**	***Intercept***
**lF**	**-1.203**	**0.001**	**12.754**	**-1.432**	**0.001**	**18.902**
**lH**	**-0.379**		**12.171**	**-0.426**		**12.634**
**hF**	**-1.188**	**0.001**	**14.704**	**-1.359**	**0.003**	**18.878**
**hH**	**-0.489**		**14.232**	**-0.181**		**12.383**
		***p* values (one sample ttest)**			***p* values (one sample ttest)**	
**lFlH**	**-0.921**	**0.012**	**14.102**	**-1.033**	**0.045**	**18.691**
**lFhH**	**-0.522**	0.246	**14.725**	**-0.236**	0.561	**13.164**
**hFlH**	**-1.225**	**0.005**	**15.014**	**-1.045**	**0.053**	**18.681**
**hFhH**	**-0.994**	**0.012**	**16.289**	**-0.923**	**0.083**	**19.772**

Table 5: Results of one sample t-test for the selectivity to faces (lF-lH, hF-hH, lFlH-lH, lFhH-hH, hFlH-lH, hFhH-hH) in ROIs along the virtual line from the FFA to PPA in the main experiment (Figure 4A and 4B). 0.001 indicates the *p* value is less than 0.001. LH is the abbreviation for the left hemisphere and RH is the abbreviation for the right hemisphere.

***p* values**	LH						RH					
	One sample ttest						One sample ttest					
ROI index	lF-lH	hF-hH	lFlH-lH	lFhH-hH	hFlH-lH	hFhH-hH	lF-lH	hF-hH	lFlH-lH	lFhH-hH	hFlH-lH	hFhH-hH
1												
2												
3												
4												
5												
6												
7												
8												
9												
10								0.006				0.007
11							0.007	0.003	0.007			0.003
12							0.003	0.001	0.002		0.005	0.001
13	0.008		0.006			0.008	0.001	0.001	0.001		0.001	0.001
14		0.009	0.003			0.001	0.001	0.001	0.001		0.001	0.001
15	0.004	0.001	0.001		0.001	0.001	0.001	0.001	0.001		0.001	0.001
16	0.001	0.001	0.001		0.001	0.001	0.001	0.001	0.001		0.001	0.001
17	0.001	0.001	0.001		0.001	0.001	0.001	0.001	0.001		0.001	0.001
18	0.006	0.007	0.001		0.001	0.001	0.001	0.001	0.001		0.001	0.001
19			0.004				0.001	0.001	0.001		0.003	0.003
20												0.004
21												
22												
23												
24												
25												
26												
27	0.002											
28	0.003	0.006										
29	0.003	0.001										
30		0.001										
31		0.001										
32		0.005										
33	0.007						0.003					
34	0.001	0.003					0.002	0.003				
35	0.001	0.001	0.002		0.001		0.003	0.001				
36	0.001	0.001	0.003		0.001		0.005	0.001				
37	0.001	0.001	0.003		0.002		0.001	0.001			0.006	
38	0.001		0.003		0.001		0.001	0.001			0.005	
39	0.002	0.001			0.003		0.001	0.001			0.008	
40	0.001	0.002			0.004		0.001	0.001				
41	0.001				0.003		0.001	0.001				
42	0.001						0.007	0.001				
43							0.008	0.001				
44							0.003	0.001				
45							0.004	0.001				
46	0.001						0.003	0.003				
47							0.003					
48												
49												
50							0.007					
51							0.007				0.004	

Table 6: Results of one sample t-test for the selectivity to houses (lH-lF, hH-hF, lFlH-lF, lFhH-lF, hFlH-hF, hFhH-hF) in the ROIs along the virtual line from the FFA to PPA in the main experiment (Figure 4C and 4D). 0.001 indicates the *p* value is less than 0.001. LH is the abbreviation for the left hemisphere and RH is the abbreviation for the right hemisphere.

***p* values**		**LH**						**RH**					
		**One sample ttest**						**One sample ttest**					
		**lH-lF**	**hH-hF**	**lFlH-lF**	**lFhH-lF**	**hFlH-hF**	**hFhH-hF**	**lH-lF**	**hH-hF**	**lFlH-lF**	**lFhH-lF**	**hFlH-hF**	**hFhH-hF**
**ROI index**	**1**												
	**2**												
	**3**												
	**4**												
	**5**												
	**6**												
	**7**												
	**8**												
	**9**												
	**10**								0.006				
	**11**							0.007	0.003		0.008		
	**12**							0.003	0.001		0.004		
	**13**	0.008						0.001	0.001		0.003		
	**14**		0.009					0.001	0.001		0.001		
	**15**	0.004	0.001					0.001	0.001		0.001		
	**16**	0.001	0.001		0.001			0.001	0.001		0.001		
	**17**	0.001	0.001		0.004			0.001	0.001		0.002		
	**18**	0.006	0.007					0.001	0.001		0.002		
	**19**							0.001	0.001		0.001		
	**20**												
	**21**												
	**22**												
	**23**												
	**24**												
	**25**												
	**26**			0.006	0.001								
	**27**	0.002		0.002	0.001								0.007
	**28**	0.003	0.006		0.001		0.006						0.008
	**29**	0.003	0.001		0.001		0.009						
	**30**		0.001		0.001		0.007			0.009			
	**31**		0.001	0.009	0.002		0.001			0.006			
	**32**		0.005	0.006	0.001		0.002			0.003	0.003		
	**33**	0.007		0.008	0.001		0.004	0.003		0.002	0.001		
	**34**	0.001	0.003	0.001	0.001		0.001	0.002	0.003	0.006	0.001		
	**35**	0.001	0.001	0.001	0.001		0.001	0.003	0.001		0.001		0.005
	**36**	0.001	0.001	0.001	0.001		0.001	0.005	0.001	0.01	0.001		0.003
	**37**	0.001	0.001	0.001	0.001		0.001	0.001	0.001	0.001	0.001		0.001
	**38**	0.001	0.001	0.003	0.001		0.002	0.001	0.001	0.001	0.001		0.001
	**39**	0.002	0.001	0.01	0.001		0.003	0.001	0.001	0.001	0.001		0.001
	**40**	0.001	0.002	0.004	0.001		0.002	0.001	0.001	0.002	0.001		0.001
	**41**	0.001		0.005	0.001		0.002	0.004	0.001		0.001		0.006
	**42**	0.001			0.001			0.007	0.001	0.01	0.001		
	**43**							0.008	0.001		0.001		
	**44**							0.003	0.001		0.001		0.002
	**45**				0.001			0.004	0.001		0.003		0.002
	**46**	0.001		0.004	0.001			0.003	0.003				
	**47**				0.007			0.003					
	**48**			0.005									
	**49**												
	**50**							0.007			0.005		
	**51**							0.007			0.005		

Table 7: Results of two-way ANOVA for face classification accuracies (2 levels of face contrast (low (lF, lFlH and lFhH) *vs.* high (hF, hFlH and hFhH)) * 3 levels of house contrast (none (lF and hF) *vs.* low (lFlH and hFlH) *vs.* high (lFhH and hFhH)), the solid red box in Figure 1B) in ROIs along the virtual line from the FFA to PPA in the main experiment (Figure 5A and 5B). 0.001 indicates the *p* value is less than 0.001. LH is the abbreviation for the left hemisphere and RH is the abbreviation for the right hemisphere.

p values		LH												
		Main effect of face contrast	Main effect of house contrast	Interaction effect	Multiple Comparisons									
					hFhH vs. hFlH	hFhH vs. hF	hFlH vs. hF	lFhH vs. lFlH	lFhH vs. lF	lFlH vs. lF	lFhH vs. hFhH	lFlH vs. hFlH	lF vs. hF
ROI index	1													
	2	0.028												
	3	0.007												
	4													
	5													
	6													
	7										0.028			
	8													
	9													
	10													
	11			0.001					0.001	0.04				
	12										0.005			
	13	0.01		0.044				0.008			0.002			
	14	0.022	0.036	0.037				0.043			0.004			
	15	0.021	0.003	0.014				0.009			0.007			
	16	0.001	0.001	0.001				0.001	0.004		0.001			
	17	0.002	0.001	0.001				0.001	0.001		0.001			
	18	0.003	0.014	0.014				0.002			0.001			
	19	0.008	0.008	0.006				0.025	0.018		0.002			
	20		0.014	0.013					0.009		0.026			
	21													
	22		0.002											
	23		0.005											
	24		0.001		0.006	0.007			0.029					
	25		0.001					0.001						
	26		0.013											
	27		0.001					0.008	0.019					
	28		0.008											
	29		0.001		0.011			0.01	0.02			0.027		
	30		0.002		0.004									
	31		0.001		0.009	0.001		0.036	0.014					
	32		0.001		0.001	0.033		0.003	0.001	0.042				
	33		0.001	0.005	0.001			0.01	0.002	0.018		0.014		
	34		0.001		0.001	0.039		0.003	0.001	0.04		0.042		
	35		0.001	0.006	0.001	0.016		0.002	0.003	0.043		0.002		
	36	0.031	0.001	0.021	0.002	0.026		0.013	0.001	0.005		0.002		
	37		0.001		0.004	0.002		0.01	0.001	0.011				
	38		0.001	0.031	0.002	0.009		0.005	0.001	0.001				
	39		0.001		0.002	0.034		0.012	0.001			0.017		
	40	0.012	0.001		0.001	0.001		0.002	0.014			0.021		
	41	0.006	0.001		0.002	0.002		0.019	0.017					
	42		0.001		0.009	0.013		0.011	0.019					
	43		0.001		0.031	0.041		0.033	0.021					
	44		0.001		0.015	0.013								
	45		0.001			0.002	0.023							
	46		0.001						0.01					
	47		0.006											
	48		0.001			0.015			0.04					
	49													
	50		0.001			0.019								
	51													

p values	RH											
				Multiple Comparisons								
ROI index	Main effect of face contrast	Main effect of house contrast	Interaction effect	hFhH vs. hFlH	hFhH vs. hF	hFlH vs. hF	lFhH vs. lFlH	lFhH vs. lF	lFlH vs. lF	lFhH vs. hFhH	lFlH vs. hFlH	lF vs. hF
1			0.01									
2												
3		0.007		0.035								
4		0.016					0.012					
5		0.001		0.034			0.001					
6		0.006	0.026									
7			0.048									
8			0.005				0.015			0.039		
9		0.005	0.006				0.011			0.018		
10	0.013	0.048										
11	0.001	0.015	0.005				0.003			0.001		
12	0.007	0.004	0.012							0.008		
13	0.009	0.001	0.001				0.006	0.001		0.003		
14	0.001	0.001	0.001				0.001	0.003		0.001		
15	0.001	0.001	0.001				0.001	0.003		0.001		
16	0.001	0.001	0.001				0.001	0.001	0.04	0.001		
17	0.002	0.001	0.001				0.001	0.001		0.001		
18	0.014	0.001	0.001				0.001	0.001	0.049	0.002		
19	0.006	0.001	0.004				0.03	0.011		0.009		
20	0.005	0.001	0.009		0.017		0.007	0.009		0.007		
21		0.02	0.001				0.019			0.017		
22		0.004	0.001				0.005	0.001		0.008		
23		0.001						0.024				
24	0.011	0.001						0.013				
25		0.003										
26		0.001	0.041					0.001				
27		0.01										
28		0.001					0.017	0.003				
29		0.001						0.032				
30		0.001	0.044	0.013	0.026			0.001	0.017		0.005	
31	0.041	0.002			0.03							
32		0.001	0.041		0.001			0.001	0.001			
33		0.001	0.026					0.001	0.008			
34		0.001	0.04	0.001	0.004			0.001	0.035			
35		0.001		0.006	0.023			0.002	0.027			
36		0.001		0.002	0.025			0.003				
37		0.001	0.037	0.002	0.003		0.045	0.002	0.008		0.035	
38	0.041	0.001		0.001	0.001			0.001	0.001			
39	0.014	0.001	0.049	0.001	0.001			0.001	0.038		0.02	
40		0.001		0.008	0.029			0.017			0.042	
41		0.001						0.012				
42		0.001						0.023				
43		0.001	0.032	0.003			0.046	0.005				
44		0.001	0.007	0.014	0.017			0.001	0.005			
45		0.001		0.001								
46		0.004	0.043	0.017							0.038	
47		0.004						0.04				
48		0.001						0.042				
49												
50												
51												

Table 8: Results of two-way ANOVA for house classification accuracies (2 levels of house contrast (low (lH, lFlH and hFlH) *vs.* high (hH, lFhH and hFhH)) * 3 levels of face contrast (none (lH and hH) *vs.* low (lFlH and lFhH) *vs.* high (hFlH and hFhH), the dashed blue box in Figure 1B) in ROIs along the virtual line from the FFA to PPA in the main experiment (Figure 5C and 5D). 0.001 indicates the *p* value is less than 0.001. LH is the abbreviation for the left hemisphere and RH is the abbreviation for the right hemisphere.

p values	LH											
	Main effect of face contrast	Main effect of house contrast	Interaction effect	Multiple Comparisons								
				hFhH vs. hFlH	lFhH vs. lFlH	lH vs. hH	lFhH vs. hFhH	hH vs. hFhH	hH vs. lFhH	lFlH vs. hFlH	lH vs. hFlH	lH vs. lFlH
ROI index 1												
2												
3												
4												
5												
6												
7												
8												
9	0.049											
10												
11												
12	0.002				0.026		0.009	0.01				
13	0.001		0.032		0.004		0.004	0.029			0.037	
14	0.001		0.002		0.021		0.008	0.045			0.016	0.006
15	0.001	0.002	0.002		0.004		0.014	0.004			0.018	0.003
16	0.001	0.001	0.001		0.001		0.001	0.001			0.001	0.001
17	0.001	0.001	0.001		0.001		0.001	0.003			0.003	0.003
18	0.001		0.001		0.001		0.003				0.042	0.008
19	0.001	0.037	0.006		0.012		0.004					
20	0.006										0.044	
21	0.004		0.049								0.008	0.04
22	0.017											
23												
24		0.004		0.003								
25	0.039	0.001	0.018		0.001							
26	0.016											
27		0.003			0.004							
28	0.006			0.038								0.006
29	0.007	0.007	0.04	0.006	0.005						0.012	
30			0.031	0.002								
31		0.013		0.005	0.018							
32		0.001		0.001	0.002							
33	0.012	0.002		0.001	0.005					0.028		
34	0.001	0.016	0.001	0.001	0.001						0.003	0.016
35	0.001	0.005	0.001	0.001	0.001					0.004	0.002	0.023
36	0.001	0.001	0.001	0.001	0.006					0.003	0.004	
37	0.001	0.009	0.001	0.002	0.005						0.004	0.004
38		0.003	0.001	0.001	0.002							
39		0.002	0.003	0.001	0.006					0.034		
40	0.036	0.001	0.006	0.001	0.001					0.042		
41		0.001		0.001	0.009							
42		0.001		0.004	0.006							
43		0.005		0.015	0.016							
44				0.008								
45		0.005										
46		0.036										
47				0.026	0.03							
48			0.016	0.028								
49												
50												
51												

p values	RH											
	Main effect of face contrast	Main effect of house contrast	Interaction effect	Multiple Comparisons								
ROI index				hFhH vs. hFlH	lFhH vs. lFlH	lH vs. hH	lFhH vs. hFhH	hH vs. hFhH	hH vs. lFhH	lFlH vs. hFlH	lH vs. hFlH	lH vs. lFlH
1												
2		0.02										
3		0.033		0.017	0.031							
4		0.009		0.037	0.006							
5		0.003		0.017	0.001							0.034
6	0.019	0.041			0.031							0.016
7												
8		0.041	0.004		0.007							
9		0.019	0.004		0.006		0.035					
10	0.04	0.008			0.042							
11	0.001		0		0.001		0.001				0.019	0.006
12	0.001		0.002		0.028		0.016					
13	0.001	0.002	0.008		0.003		0.005	0.001			0.013	0.013
14	0.001	0.006	0.001		0.001		0.001	0.001			0.002	0.001
15	0.001	0.001	0.001		0.001		0.001	0.001			0.001	0.001
16	0.001	0.002	0.001		0.001		0.001	0.001			0.001	0.001
17	0.001	0.002	0.001		0.001		0.001	0.001			0.001	0.001
18	0.001	0.001	0.001		0.001		0.003	0.002			0.003	0.008
19	0.001		0.003		0.015		0.018	0.027			0.021	
20	0.001		0.002		0.003		0.013				0.001	0.002
21	0.003	0.045	0.041		0.009		0.033					
22	0.001	0.047	0.018		0.003		0.016				0.039	0.009
23			0.038									
24												
25												
26		0.002										
27												
28					0.008							
29		0.011			0.045							
30	0.022	0.006		0.006						0.01		
31			0.014								0.042	
32	0.048	0.04										
33												
34		0.005	0.001	0.001							0.024	
35		0.006		0.003								
36	0.002	0.03	0.005	0.001							0.002	0.014
37	0.023	0.001		0.001	0.023							
38	0.009	0.014		0.001								
39	0.001	0.002	0.02	0.001						0.041	0.014	
40		0.008		0.004								
41		0.049									0.014	
42		0.029									0.041	
43		0.001		0.001	0.023							
44	0.02	0.009	0.02	0.007							0.016	
45		0.001		0.001								
46		0.048		0.009								
47												
48												
49		0.041										
50												
51		0.033										

Table 9: Results of two-way ANOVA for responses to faces (2 levels of face contrast (low (lF, lFlH and lFhH) *vs.* high (hF, hFlH and hFhH)) * 3 levels of house contrast (none (lF and hF) *vs.* low (lFlH and hFlH) *vs.* high (lFhH and hFhH)), the solid red box in the Figure 1B) in ROIs along the virtual line from the occipital pole to the FFA in the main experiment (Figure 6A and 6B). 0.001 indicates the *p* value is less than 0.001. LH is the abbreviation for the left hemisphere and RH is the abbreviation for the right hemisphere.

p values	from occipital to FFA (LH)											
	Main effect of face contrast	Main effect of house contrast	Interaction effect	Multiple Comparisons								
ROI index				hFhH vs. hFlH	hFhH vs. hF	hFlH vs. hF	lFhH vs. lFlH	lFhH vs. lF	lFlH vs. lF	lFhH vs. hFhH	lFlH vs. hFlH	lF vs. hF
1	0.031	0.008							0.037			
2	0.031	0.011							0.041			
3	0.025	0.012										
4	0.025	0.01										
5	0.02	0.005										
6	0.011	0.002		0.013	0.027							
7	0.015	0.001	0.043	0.008	0.014							
8	0.01	0.001	0.035	0.006	0.008			0.028				0.028
9	0.005	0.001	0.038	0.007	0.005		0.047	0.018				0.024
10	0.004	0.001	0.044	0.009	0.005		0.008	0.013				0.022
11	0.01	0.001	0.031	0.019	0.002		0.006	0.009				0.026
12	0.032	0.001	0.011	0.015	0.006		0.008	0.003				
13	0.045	0.001	0.011				0.046	0.008				
14		0.001	0.036					0.016				
15		0.001			0.027			0.007	0.017			
16		0.001		0.023	0.028				0.021			
17		0.048										
18												
19												
20		0.006	0.001	0.04								
21		0.002	0.001	0.009					0.033	0.008		
22		0.001	0.002	0.01	0.026				0.017	0.007		
23		0.002	0.001		0.022				0.012	0.002		
24		0.039	0.001		0.027				0.043	0.001		
25		0.004	0.001	0.002	0.002				0.049	0.002		
26		0.029	0.001							0.008		
27	0.03	0.006	0.006						0.049	0.021		0.025
28	0.039	0.003			0.013							
29		0.037	0.03									
30		0.047	0.01	0.015						0.012		
31			0.009							0.019		
32			0.031									
33												
34												
35												
36												
37												
38		0.02										
39		0.001			0.02							
40		0.001			0.035							
41		0.002										
42		0.001										
43		0.027										
44	0.032		0.016							0.003		
45	0.003	0.005	0.001				0.001	0.013		0.001		
46	0.001	0.002	0.001				0.003	0.005		0.001		
47	0.001	0.013	0.001				0.028	0.037		0.001		
48	0.007	0.031	0.004							0.01		
49	0.003	0.01	0.001				0.033	0.036		0.005		
50	0.002	0.027	0.004							0.014		
51	0.008		0.004							0.007		
52	0.045		0.01							0.023		
53												
54												
55												
56												
57									0.049			
58			0.014									
59			0.008				0.031			0.015		
60												

p values	from occipital to FFA (RH)											
	Main effect of face contrast	Main effect of house contrast	Interaction effect	Multiple Comparisons								
ROI index				hFhH vs. hFlH	hFhH vs. hF	hFlH vs. hF	lFhH vs. lFlH	lFhH vs. lF	lFlH vs. lF	lFhH vs. hFhH	lFlH vs. hFlH	lF vs. hF
1	0.005	0.001		0.02	0.007							0.034
2	0.018	0.001		0.016	0.01							0.047
3	0.027	0.001		0.011	0.009							
4	0.032	0.001		0.012	0.008							
5	0.03	0.001		0.02	0.007							
6	0.036	0.001		0.015	0.014							
7	0.027	0.002		0.01	0.018							
8	0.026	0.004		0.02	0.034							
9	0.016	0.012			0.04							
10		0.003										
11		0.001					0.044	0.024				
12		0.001					0.034	0.028				
13	0.007	0.001			0.045		0.008	0.048				
14	0.01	0.001			0.014							
15	0.02	0.001			0.005					0.021		
16	0.019	0.001		0.02	0.001			0.018	0.002	0.03		0.034
17	0.037	0.001			0.002				0.038			
18		0.04										
19												
20												
21		0.015							0.011			
22		0.016	0.004						0.001			
23		0.002	0.013		0.006				0.018			
24		0.001	0.003		0.003			0.029	0.002			
25		0.001	0.013		0.013			0.035	0.049			
26		0.001			0.027							
27		0.014	0.008	0.007				0.041				
28		0.042	0.002									
29		0.032	0.019		0.031							
30		0.004						0.004				
31		0.048	0.026					0.035				
32		0.021							0.035			
33												
34												
35												
36												
37												
38												
39												
40			0.023			0.023				0.034		
41	0.025		0.042									
42		0.025	0.003				0.008			0.019		
43		0.001	0.001				0.003	0.015		0.004		
44	0.028	0.001	0.001				0.008	0.02		0.005		
45	0.008	0.001	0.001				0.003	0.005		0.002		
46	0.001	0.001	0.001				0.001	0.001		0.001		
47	0.001	0.001	0.001				0.002	0.001		0.001		
48	0.002	0.001	0.001		0.035		0.006	0.001		0.001		
49	0.016	0.003	0.016				0.001	0.038		0.001		
50	0.016	0.026								0.015		
51	0.036											
52	0.043											
53												
54												
55												
56												
57												
58												
59	0.042											
60	0.033											

Table 10: Results of two-way ANOVA for responses to houses (2 levels of house contrast (low (lH, lFlH and hFlH) *vs.* high (hH, lFhH and hFhH)) * 3 levels of face contrast (none (lH and hH) *vs.* low (lFlH and lFhH) *vs.* high (hFlH and hFhH), the dashed blue box in the Figure 1B) in ROIs along the virtual line from the occipital pole to the PPA in the main experiment (Figure 6C and 6D). 0.001 indicates the *p* value is less than 0.001. LH is the abbreviation for the left hemisphere and RH is the abbreviation for the right hemisphere.

p values		from occipital to PPA (LH)											
		Main effect of face contrast	Main effect of house contrast	Interaction effect	Multiple Comparisons								
					hFhH vs. hFlH	lFhH vs. lFlH	lH vs. hH	lFhH vs. hFhH	hH vs. hFhH	hH vs. lFhH	lFlH vs. hFlH	lH vs. hFlH	lH vs. lFlH
ROI index	1				0.02								
	2		0.041		0.011								
	3		0.012		0.007			0.034					
	4	0.036	0.001		0.006								
	5	0.043	0.001		0.005								
	6		0.001		0.007		0.038						
	7	0.044	0.001		0.005		0.023						
	8	0.03	0.002		0.009	0.029	0.03						
	9	0.01	0.006		0.017	0.038							
	10	0.024	0.01		0.026	0.026							
	11		0.017			0.047							
	12		0.013										
	13		0.008				0.044						
	14		0.001			0.008	0.019						
	15		0.001		0.011		0.002						
	16		0.001		0.011		0.034						
	17		0.011										
	18	0.038											
	19		0.014										
	20												
	21		0.032		0.005								
	22			0.011	0.004								
	23			0.033									
	24				0.008						0.019		
	25												
	26												
	27												
	28			0.019									
	29			0.012									
	30			0.021	0.021								
	31			0.004	0.007								
	32			0.008	0.013								
	33			0.011	0.032								
	34			0.031									
	35												
	36												
	37												
	38			0.041	0.028								
	39		0.005	0.036	0.017								
	40		0.006		0.046								
	41		0.005		0.045								
	42		0.008	0.009	0.018								
	43		0.002	0.007	0.01								
	44	0.005	0.001	0.001	0.001	0.004	0.031				0.035	0.038	
	45	0.001	0.001	0.001	0.001	0.001	0.024				0.034	0.007	
	46	0.001	0.001	0.004	0.001	0.001	0.047					0.01	0.017
	47	0.001	0.001		0.009	0.001						0.01	0.003
	48	0.001	0.006		0.033	0.022						0.019	0.007
	49	0.007	0.04										
	50												
	51	0.011											
	52	0.049											
	53												
	54		0.034		0.035								
	55	0.008	0.049			0.049							
	56	0.009											
	57	0.014											
	58	0.011						0.047					
	59		0.006										
	60		0.024										

p values		from occipital to PPA (RH)											
		Main effect of face contrast	Main effect of house contrast	Interaction effect	Multiple Comparisons								
					hFhH vs. hFlH	lFhH vs. lFlH	lH vs. hH	lFhH vs. hFhH	hH vs. hFhH	hH vs. lFhH	lFlH vs. hFlH	lH vs. hFlH	lH vs. lFlH
ROI index	1	0.001	0.004	0.006			0.005					0.006	
	2	0.001	0.004	0.005			0.004					0.006	
	3	0.001	0.004	0.006			0.005					0.005	
	4	0.002	0.003	0.01			0.007					0.009	
	5	0.004	0.001	0.011			0.007					0.014	
	6	0.008	0.008	0.007			0.009					0.019	
	7	0.008	0.009	0.011			0.01					0.016	
	8	0.022	0.01	0.023			0.013					0.024	0.037
	9	0.035	0.005				0.013						0.043
	10	0.027	0.005			0.009	0.013						
	11	0.017	0.003			0.003	0.008						
	12	0.034	0.001			0.01	0.001						
	13		0.003			0.025	0.031						
	14	0.011	0.002					0.023					
	15	0.002	0.003		0.034			0.009	0.025				
	16	0.003	0.017		0.01				0.036				
	17	0.011	0.017		0.02								0.032
	18		0.03				0.008						
	19						0.037						
	20												
	21		0.048		0.04								
	22		0.01	0.024	0.015								
	23				0.015								
	24		0.036	0.015	0.001								
	25			0.005	0.005								
	26			0.035	0.022								
	27			0.025	0.019								
	28		0.04		0.044								
	29		0.005	0.047	0.005								
	30				0.012								
	31			0.007	0.048								
	32			0.001	0.005								
	33		0.045	0.008	0.007								
	34		0.003	0.003	0.002								
	35		0.002	0.006	0.003								
	36		0.002	0.016	0.01								
	37		0.004	0.039	0.023								
	38		0.011										
	39		0.011		0.046								
	40		0.009										
	41	0.026	0.006										
	42		0.023										
	43	0.033	0.003				0.002						
	44	0.004	0.001		0.02		0.001						
	45	0.004	0.001		0.02	0.024	0.011						
	46	0.001	0.001		0.008	0.015	0.015					0.038	
	47	0.005	0.001		0.013	0.008	0.037						
	48	0.016	0.001		0.029	0.02	0.041						
	49	0.007	0.007	0.049									
	50	0.004	0.003									0.035	
	51		0.019										
	52		0.03										
	53		0.018										
	54		0.038										
	55												
	56												
	57												
	58												
	59												
	60												

Table 11: Results of two-way ANOVA for face classification accuracies (2 levels of face contrast (low (lF, lFlH and lFhH) *vs.* high (hF, hFlH and hFhH)) * 3 levels of house contrast (none (lF and hF) *vs.* low (lFlH and hFlH) *vs.* high (lFhH and hFhH)), the solid red box in the Figure 1B) in ROIs along the virtual line from the occipital pole to the FFA in the main experiment (Figure 6E and 6F). 0.001 indicates the *p* value is less than 0.001. LH is the abbreviation for the left hemisphere and RH is the abbreviation for the right hemisphere.

p values	from occipital to FFA (LH)											
				Multiple Comparisons								
ROI index	Main effect of face contrast	Main effect of house contrast	Interaction effect	hFhH vs. hFlH	hFhH vs. hF	hFlH vs. hF	lFhH vs. lFlH	lFhH vs. lF	lFlH vs. lF	lFhH vs. hFhH	lFlH vs. hFlH	lF vs. hF
1												
2												
3												
4		0.01										
5		0.027										
6		0.001			0.028							
7		0.002			0.02							
8		0.002			0.018							
9												
10												
11	0.05	0.002						0.032	0.017			
12		0.001						0.001	0.011			
13		0.015						0.027				
14		0.001			0.008			0.013				
15		0.046										
16		0.004										
17												
18												
19												
20											0.005	
21												
22		0.004		0.002				0.004				
23												
24												
25		0.02						0.048				
26		0.02										
27		0.001									0.024	
28			0.045									
29		0.022										
30		0.006						0.007				
31		0.004						0.04				
32		0.026						0.01				
33												
34												
35	0.006	0.001					0.036	0.019		0.043		
36		0.001						0.016				
37	0.011	0.001						0.045		0.003		
38		0.001						0.009	0.044			
39	0.022	0.035										
40	0.012											
41			0.021									
42												
43		0.001			0.045							
44		0.001	0.001				0.002	0.002		0.005		
45	0.012	0.001	0.026				0.001	0.015		0.007		
46	0.001	0.001	0.001				0.001	0.004		0.001		
47	0.001	0.001	0.001				0.001	0.008		0.001		
48	0.008	0.002					0.011			0.01		
49		0.004	0.029				0.04			0.046		
50												
51												
52												
53												
54												
55												
56												
57												
58												
59												
60												

p values		from occipital to FFA (RH)											
		Main effect of face contrast	Main effect of house contrast	Interaction effect	Multiple Comparisons								
					hFhH vs. hFlH	hFhH vs. hF	hFlH vs. hF	lFhH vs. lFlH	lFhH vs. lF	lFlH vs. lF	lFhH vs. hFhH	lFlH vs. hFlH	lF vs. hF
ROI index	1												
	2	0.016											
	3												
	4												
	5												
	6												
	7												
	8												
	9												
	10												
	11		0.041										
	12												
	13		0.032										
	14												
	15							0.036					
	16												
	17					0.043							
	18												
	19			0.046									
	20			0.005									0.036
	21		0.002	0.007	0.022				0.048	0.012			
	22												
	23												
	24												
	25		0.002	0.023	0.008				0.02				
	26	0.002	0.001			0.018						0.005	
	27			0.009								0.014	
	28	0.022											
	29		0.001						0.003				
	30		0.004										
	31												
	32		0.02										
	33												
	34	0.042											
	35												
	36												
	37		0.001						0.011	0.019			
	38												
	39												
	40	0.029											
	41		0.002										
	42		0.001	0.004				0.001	0.006		0.004		
	43		0.004	0.028				0.014	0.019				
	44		0.001	0.001				0.001	0.002		0.009		
	45	0.002	0.001	0.001				0.001	0.001	0.036	0.001		
	46	0.002	0.001	0.001				0.001	0.001	0.003	0.001		
	47	0.001	0.001	0.001				0.001	0.001		0.001		
	48	0.021	0.001	0.001				0.001	0.001		0.001		
	49	0.028	0.001	0.015		0.023		0.002	0.001		0.006		
	50		0.001	0.019					0.021		0.029		
	51	0.035	0.011										
	52		0.004	0.003					0.016			0.005	
	53		0.043										
	54												
	55												
	56												
	57												
	58												
	59		0.012				0.005						
	60												

Table 12: Results of two-way ANOVA for house classification accuracies (2 levels of house contrast (low (lH, lFlH and hFlH) *vs.* high (hH, lFhH and hFhH)) * 3 levels of face contrast (none (lH and hH) *vs.* low (lFlH and lFhH) *vs.* high (hFlH and hFhH), the dashed blue box in the Figure 1B) in ROIs along the virtual line from the occipital pole to the PPA in the main experiment (Figure 6G and 6H). 0.001 indicates the *p* value is less than 0.001. LH is the abbreviation for the left hemisphere and RH is the abbreviation for the right hemisphere.

p values		from occipital to PPA (LH)											
		Main effect of face contrast	Main effect of house contrast	Interaction effect	Multiple Comparisons								
					hFhH vs. hFlH	lFhH vs. lFlH	lH vs. hH	lFhH vs. hFhH	hH vs. hFhH	hH vs. lFhH	lFlH vs. hFlH	lH vs. hFlH	lH vs. lFlH
ROI index	1												
	2												
	3												
	4			0.029									
	5			0.007									
	6			0.001	0.007								
	7												
	8												
	9												
	10												
	11												
	12		0.032			0.027							
	13		0.022										
	14		0.018		0.003								
	15												
	16												
	17												
	18												
	19												
	20	0.028											
	21												
	22			0.009									
	23		0.013	0.016							0.009		
	24												
	25												
	26												0.038
	27												
	28			0.032	0.018								
	29												
	30												
	31		0.004		0.019								
	32												
	33				0.017								
	34												
	35		0.019										
	36		0.004		0.006								
	37		0.007										
	38												
	39	0.029	0.008		0.036								
	40		0.002			0.028							
	41		0.001		0.028	0.04							
	42		0.02										
	43												
	44		0.016	0.001	0.003	0.042							
	45		0.001	0.001	0.001	0.001					0.028	0.022	
	46	0.001	0.001	0.001	0.004	0.002						0.003	0.004
	47	0.001	0.001	0.001	0.001	0.001					0.02	0.002	0.018
	48	0.037	0.002	0.002	0.003	0.001							
	49		0.005										
	50	0.011	0.006										
	51	0.034	0.005		0.017								
	52	0.001	0.041						0.02				
	53	0.013											
	54												
	55												
	56												
	57												
	58												
	59		0.02										
	60		0.035										

p values	from occipital to PPA (RH)											
	Main effect of face contrast	Main effect of house contrast	Interaction effect	Multiple Comparisons								
ROI index				hFhH vs. hFlH	lFhH vs. lFlH	lH vs. hH	lFhH vs. hFhH	hH vs. hFhH	hH vs. lFhH	lFlH vs. hFlH	lH vs. hFlH	lH vs. lFlH
1					0.003		0.011					
2					0.027							
3												
4												
5												
6					0.042							
7												
8												
9												
10												
11												
12												
13		0.017		0.047								
14												
15												
16												
17												
18												
19												
20												
21												
22										0.017		
23												
24												
25			0.049									
26												
27												
28												
29												
30												
31			0.025									
32			0.006	0.013								
33			0.005	0.004								
34			0.008							0.017		
35												
36			0.001	0.001								
37		0.023		0.037								
38			0.04	0.033	0.011							
39	0.006		0.044									
40		0.005										
41		0.011	0.038	0.001						0.014	0.033	
42		0.004		0.023								
43		0.01		0.004								
44		0.001		0.001								
45	0.005											
46	0.001	0.001		0.001	0.022						0.035	
47	0.016	0.002		0.02	0.021							
48		0.001		0.001	0.004							
49				0.018								
50	0.001		0.029								0.001	0.002
51												
52			0.006								0.034	
53					0.041							
54					0.014							
55		0.045										
56												
57												
58												
59												
60		0.044										

Table 13: Coordinates of the FFA peak foci (16th.), the PPA peak foci (36th.) and two endpoints (the 1st. and the 51th.) of the specified virtual line connecting the FFA and PPA for each subject. LH is the abbreviation for the left hemisphere and RH is the abbreviation for the right hemisphere.

subject index	1st. (start point) (x, y, z)			16th. (FFA peak foci) (x, y, z)			36th. (PPA peak foci) (x, y, z)			51th. (end point) (x, y, z)		
**LH**												
1	-42	-36	-60	-20	-63	-56	8	-98	-51	29	-121	-51
2	-39	-77	-67	-20	-87	-68	16	-91	-56	28	-96	-41
3	-33	-106	-50	-21	-105	-55	3	-98	-51	13	-94	-43
4	-23	-95	-54	-13	-84	-59	13	-60	-49	22	-44	-36
5	-24	-101	-57	-13	-97	-62	12	-84	-60	21	-77	-53
6	-31	-77	-68	-16	-81	-65	4	-87	-57	16	-91	-51
7	-31	-83	-37	-26	-75	-50	2	-49	-53	9	-32	-46
8	-23	-88	-57	-7	-92	-55	18	-93	-45	28	-96	-34
9	-27	-111	-55	-17	-97	-62	7	-74	-55	14	-59	-43
10	-20	-115	-40	-15	-102	-53	19	-73	-46	33	-49	-34
11	-26	-92	-49	-16	-80	-57	6	-54	-50	19	-35	-41
12	-27	-78	-52	-13	-76	-55	8	-71	-52	18	-68	-44
**RH**												
1	29	-73	-53	13	-79	-53	-15	-78	-38	-26	-79	-24
2	33	-97	-61	19	-92	-70	-12	-70	-65	-22	-53	-52
3	47	-86	-53	32	-96	-57	-9	-95	-39	-20	-100	-22
4	28	-78	-53	19	-75	-56	5	-67	-52	-5	-60	-48
5	37	-74	-66	18	-83	-65	-13	-90	-55	-27	-96	-44
6	49	-66	-68	34	-71	-72	1	-71	-66	-13	-72	-54
7	33	-58	-59	22	-56	-63	3	-50	-60	-7	-45	-55
8	29	-109	-45	15	-108	-49	-11	-103	-44	-22	-102	-36
9	45	-58	-66	28	-77	-62	3	-98	-52	-14	-112	-44
10	29	-90	-57	20	-89	-60	7	-87	-61	-1	-84	-59
11	33	-63	-58	16	-60	-62	-10	-48	-50	-22	-36	-34
12	32	-98	-39	26	-84	-52	4	-56	-58	-3	-37	-56

Table 14: The regression slope coefficients of averaged beta values for face-only conditions (lF, hF) and house-only conditions (lH, hH) across the selected ROIs from the FFA to PPA for each subject. LH is the abbreviation for the left hemisphere and RH is the abbreviation for the right hemisphere.

subject index	ROI 1-10 _lF	ROI 1-10 _hF	ROI 21-30 _lF	ROI 21-30 _hF	ROI 21-30 _lH	ROI 21-30 _hH	ROI 41-51 _lH	ROI 41-51 _hH
LH								
1	0.217	0.200	-0.745	-0.875	-0.799	-0.874	0.374	0.104
2	0.197	0.693	-2.651	-2.560	-0.456	-0.863	-1.345	-1.412
3	0.003	0.561	-0.773	-0.455	-0.559	-0.967	0.756	0.853
4	0.396	0.327	-0.254	-0.062	0.464	0.926	-1.008	-2.201
5	0.308	-0.079	-3.189	-4.044	-3.268	-2.990	-1.592	-1.768
6	0.308	0.664	-0.620	-0.951	-0.087	-1.657	-0.797	-0.880
7	0.453	-0.307	-1.541	-1.505	1.073	1.077	-0.837	-1.115
8	0.310	1.074	-0.933	-0.887	-1.172	-0.384	-0.006	1.104
9	0.587	1.809	-0.787	-0.812	0.002	-0.487	-0.164	-0.450
10	1.400	0.726	-0.853	-0.279	-0.513	-0.601	0.533	1.229
11	-0.285	0.612	-0.223	-0.038	0.601	0.599	-1.922	-1.937
12	0.423	0.644	-1.866	-1.789	0.160	0.350	-0.061	-0.381
mean	0.360	0.577	-1.203	-1.188	-0.379	-0.489	-0.506	-0.571
RH								
1	0.226	2.178	-5.398	-5.264	-2.747	-3.295	-1.500	-1.296
2	-0.141	-0.595	-3.386	-3.291	-0.973	-1.054	-1.610	-1.989
3	-0.023	0.720	-0.378	-0.413	-0.714	-0.522	-1.616	-1.186
4	1.663	2.038	-0.415	-0.497	0.322	0.217	-0.076	-0.511
5	2.476	2.089	-1.853	-2.033	-0.593	-0.395	-1.420	-2.216
6	0.284	0.526	-0.226	0.535	1.104	1.298	-0.720	-1.041
7	0.053	0.441	-1.062	-0.704	0.752	1.094	0.931	0.785
8	0.879	0.700	-1.992	-1.340	-2.302	-2.150	-4.401	-3.905
9	1.272	1.692	-0.361	-0.691	-1.676	0.961	0.636	0.063
10	0.778	0.631	0.172	-0.276	1.058	1.236	-0.035	-0.553
11	0.133	0.139	-2.096	-2.602	-1.068	-1.334	-0.022	-0.518
12	0.070	0.502	-0.194	0.256	1.719	1.777	0.591	0.649
mean	0.639	0.922	-1.433	-1.360	-0.426	-0.181	-0.770	-0.977

Figure S1: Schematic visualization of the defined virtual line connecting the FFA and PPA on a flattened gray matter cortex from 5 representative subjects. Each region was defined individually from the functional localizer scan of each participant. The FFA (yellow), PPA (cyan) and the virtual line (red) connecting the FFA and PPA are shown on flattened cortices. LH is the abbreviation for the left hemisphere and RH is the abbreviation for the right hemisphere.

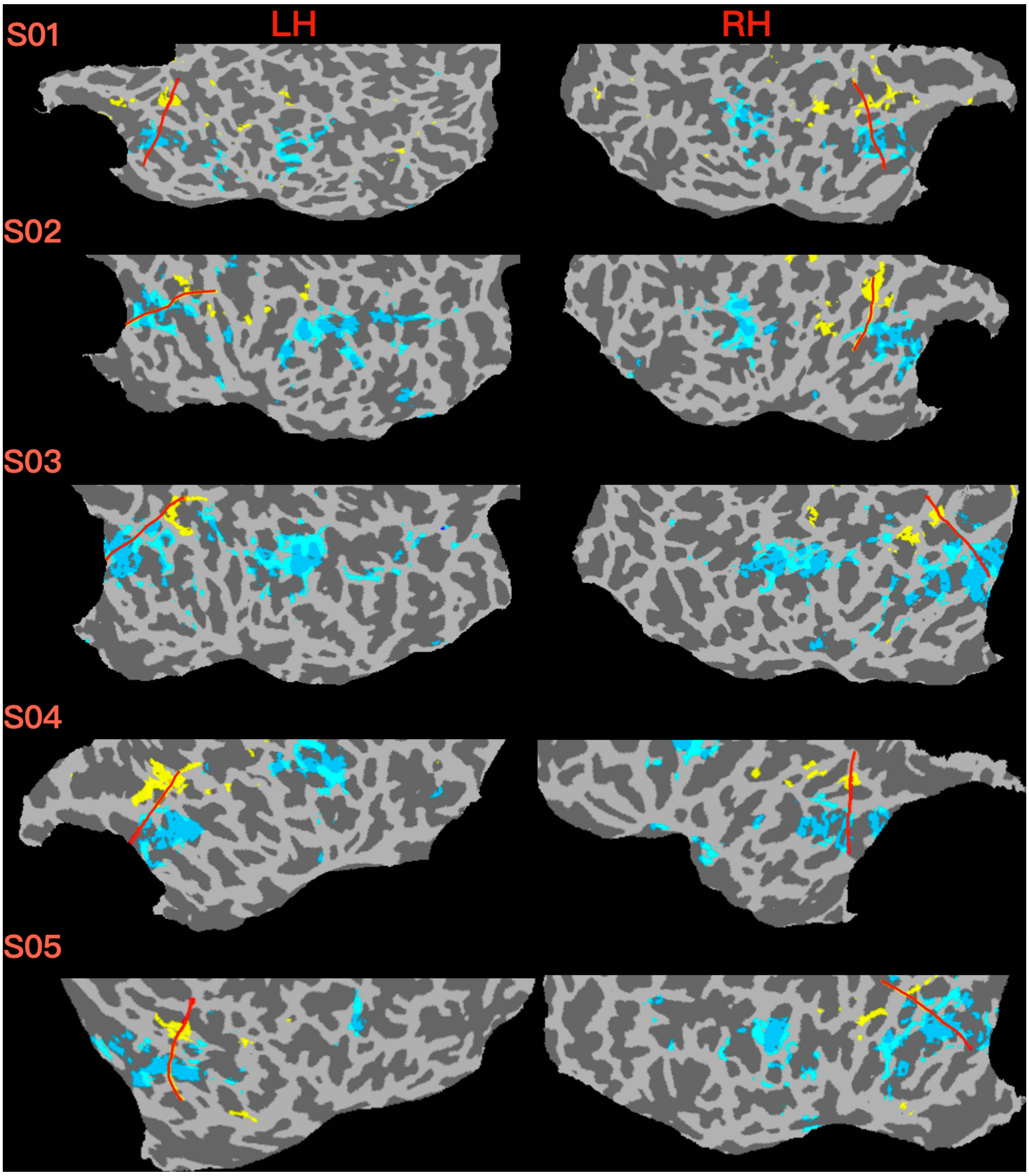

Figure S2: Univariate and multivariate results of the functional localizer scan along virtual lines from the occipital pole to the FFA and from the occipital pole to the PPA. ***A, B*** Responses to faces and houses across ROIs along the virtual line from the occipital pole to the FFA. ***C, D*** Responses to faces and houses across ROIs along the virtual line from the occipital pole to the PPA. The solid red line represents the face condition and the dashed black line represents the house condition. ***E,*** Results of classification accuracies for discriminating faces versus houses across ROIs along the virtual line from the occipital pole to the FFA. ***F,*** Results of classification accuracies across ROIs along the virtual line from the occipital pole to the PPA. The solid red line represents the left hemisphere and the dashed black line represents the right hemisphere. LH is the abbreviation for the left hemisphere, and RH is the abbreviation for the right hemisphere.

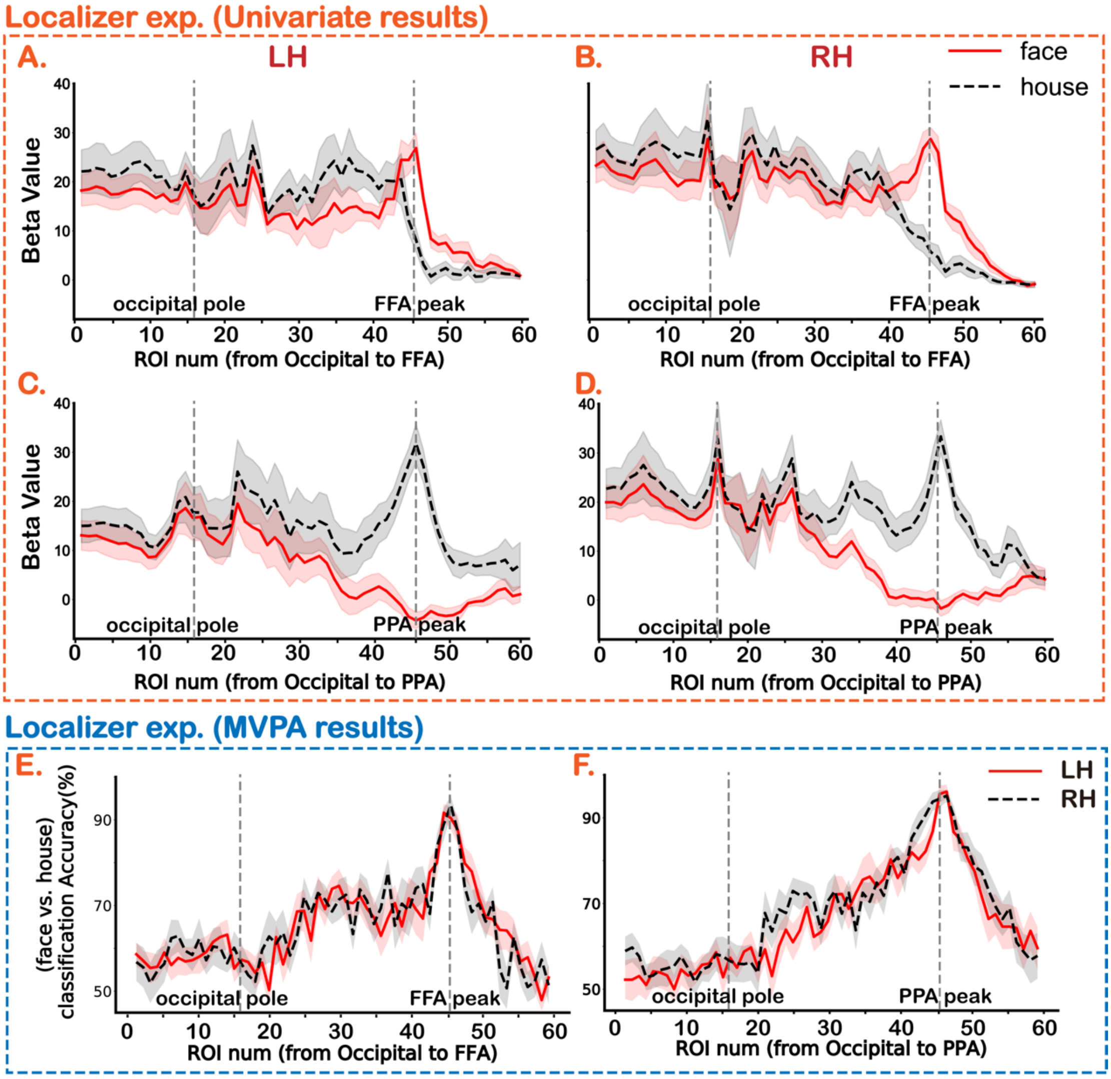

Figure S3: Group analysis of surface-based searchlight classification accuracy for discriminating faces versus houses for each hemisphere. Colored vertices indicate searchlight clusters with significantly above-chance level classification accuracy (chance level = 0.5, $p < 0.001$, uncorrected, red/yellow). LH is the abbreviation for the left hemisphere, and RH is the abbreviation for the right hemisphere.

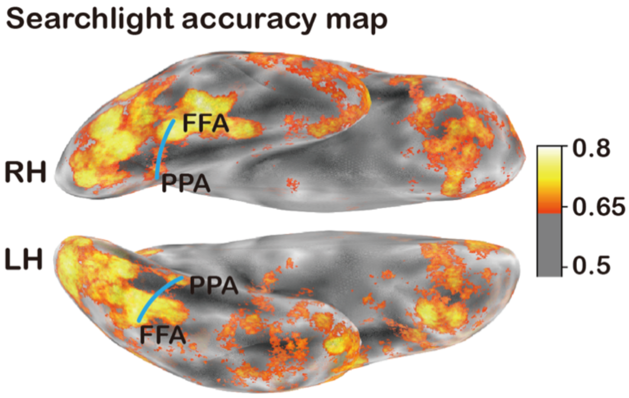

Supplement: Supplementary file 1 — Supplementary Tables [file BRB3-12-e2706-s001.pdf]
